# Supplementary material for: Precise synthesis of sulfur-containing polymers via cooperative dual organocatalysts with high activity
Source: Nat Commun. 2018 May 30;9:2137. doi: 10.1038/s41467-018-04554-5 (PMC5976647; doi:10.1038/s41467-018-04554-5)
Supplement: Supplementary file 1 — Supplementary Information [file 41467_2018_4554_MOESM1_ESM.pdf]

Zhang et al. Precise synthesis of sulfur-containing polymers via cooperative dual organocatalysts with high activity

Table of Contents

Supplementary Table 1. COS/PO copolymerization catalyzed by organic bases and TU-1/organic base pairs at 25°C **5**

Supplementary Table 2. COS/PO copolymerization catalyzed by TU-1/DBU at different temperature. **6**

Supplementary Table 3. Copolymerization of COS with PO in the presence of different amounts of BnOH **7**

Supplementary Table 4. Copolymerization of COS with PO in the presence of different amounts of H_2O **7**

Supplementary Figure 1. The photos of (a) PO/COS, (b) PGE/COS, (c) CHO/COS copolymers **8**

Supplementary Figure 2. (a)^{1}H NMR spectrum of the crude product of entry 2, Table 1; (b) ^{1}H NMR spectrum of the purified product of entry 2, Table 1; (c) ^{13}C NMR spectrum of the purified product of entry 2, Table 1 .... **9**

Supplementary Figure 3. (a)^{1}H NMR spectrum of the crude product of entry 9, Table 1; (b) ^{1}H NMR spectrum of the purified product of entry 9, Table 1; (c) ^{13}C NMR spectrum of the purified product of entry 9, Table 1 .... **10**

Supplementary Figure 4. (a)^{1}H NMR spectrum of the crude product of entry 10, Table 1; (b) ^{1}H NMR spectrum of the purified product of entry 10, Table 1; (c) ^{13}C NMR spectrum of the purified product of entry 10, Table 1 .... **11**

Supplementary Figure 5. (a)^{1}H NMR spectrum of the crude product of entry 11, Table 1; (b) ^{1}H NMR spectrum of the purified product of entry 11, Table 1; (c) ^{13}C NMR spectrum of the purified product of entry 11, Table 1 .... **12**

Supplementary Figure 6. (a)^{1}H NMR spectrum of the crude product of entry 12, Table 1; (b) ^{1}H NMR spectrum of the purified product of entry 12, Table 1; (c) ^{13}C NMR spectrum of the purified product of entry 12, Table 1 .... **13**

Supplementary Figure 7. (a)^{1}H NMR spectrum of the crude product of entry 13, Table 1; (b) ^{1}H NMR spectrum of the purified product of entry 13, Table 1; (c) ^{13}C NMR spectrum of the purified product of entry 13, Table 1 .... **14**

Supplementary Figure 8. (a)^{1}H NMR spectrum of the crude product of entry 15, Table 1; (b) ^{1}H NMR spectrum of the purified product of entry 15, Table 1; (c) ^{13}C NMR spectrum of the purified product of entry 15, Table 1 .... **15**

Supplementary Figure 9. (a)^{1}H NMR spectrum of the crude product of entry 16, Table 1; (b) ^{1}H NMR spectrum of the purified product of entry 16, Table 1; (c) ^{13}C NMR spectrum of the purified product of entry 16, Table 1 .... **16**

Supplementary Figure 10. (a)^{1}H NMR spectrum of the crude product of entry 17, Table 1; (b) ^{1}H NMR spectrum of the purified product of entry 17, Table 1; (c) ^{13}C NMR spectrum of the purified product of entry 17, Table 1 .... **17**

Supplementary Figure 11. (a)^{1}H NMR spectrum of the crude product of entry 18, Table 1; (b) ^{1}H NMR spectrum of the purified product of entry 18, Table 1; (c) ^{13}C NMR spectrum of the purified product of entry 18, Table 1 .... **18**

Supplementary Figure 12. ^{1}H NMR spectra of crude products in the control experiment that denied the possible route of ring-opening polymerization of the cyclic monothiocarbonate firstly formed **19**

Supplementary Figure 13. PO: DBU: TU-1 of 100: 1: 1 was performed for 3.5 h at 25°C, (a) 600 MHz ^{1}H NMR spectra, (b) 125 MHz ^{13}C NMR spectra; PO: DBU: TU-1: BnOH of 100: 1: 1: 1 was performed for 3.5 h at 25°C, (c) 600 MHz ^{1}H NMR spectra, (d) 125 MHz ^{13}C NMR spectra..20

Supplementary Figure 14. (a) GPC curves when [DBU] = [TU-1] = 0.2 M; (b) GPC curves when [DBU] = [TU-1] = 0.2 M, [BnOH] = 0.08M 21

Supplementary Figure 15. (a) Zero-order kinetic plots for copolymerization of COS and PO when [DBU] = [TU-1], (b) Zero-order kinetic plots for copolymerization of COS and PO when [DBU] = [TU-1], [BnOH] = 0.08 M, (c) Zero-order kinetic plots for copolymerization of COS and PO when [**P2**] = [TU-1]……..22

Supplementary Figure 16. ^{1}H NMR spectrum of the purified product of entry 5 in Table S3. 23

Supplementary Figure 17. GPC curves of entries 1-5 in Table S3 (small shoulder peak at high molecular weight was caused by trace water). 23

Supplementary Figure 18. GPC curves (black: entry 1 in Table S3; red: 0.4 mol% H_2O was added under the same conditions with entry 1 in Table S3) 23

Supplementary Figure 19. ^{1}H NMR spectrum of the purified product of entry 5 in Table S4. 24

Supplementary Figure 20. GPC curves of entries 1-4 in Table S4. 24

Supplementary Figure 21. The effect of H_2O content on the molecular weights of the COS/PO copolymers (Table S4) 24

Supplementary Figure 22. MALDI-TOF MS spectra of PPTMCs in entry 3 in Table S4. 25

Supplementary Figure 23. Chain extension reaction. 26

Supplementary Figure 24. ^{1}H NMR spectra of (a) the chemical shift of the *ortho*-protons of TU-1 with different concentration of [**P2**] = [TU-1] in $CDCl_3$, (b) he chemical shift of the *ortho*-protons of TU-1 with different concentration of [DBU] = [TU-1] in $CDCl_3$ 27

Supplementary Figure 25 ^{1}H NMR spectra of (a) DBU, (b) TU-1, (c) TU-1/DBU (1/1), (d) TU-1/DBU/COS (1/1/excess), (e) DBU/COS (1/excess), (f) TU-1/DBU/PO (1/1/1), and (g) TU-1/DBU/PO/COS (1/1/1/excess). 0.5 M TU-1 (DBU) in $CDCl_3$………………..…………………….28

Supplementary Figure 26. ^{1}H NMR spectra of (a) BnOH, BnOH/DBU (1/1), (b) BnOH/DBU/COS (1/1/excess), 72% BnO^- was equipped with COS, 0.5M [BnOH] ([DBU]) in $CDCl_3$ ……………… …29

Supplementary Figure 27. ^{1}H NMR spectra of (a) TU-2, (b) TU-3 in $CDCl_3$ 30

Supplementary Figure 28. ^{1}H NMR spectra of (a) DBU, TU-2, DBU/TU-2 (1/1), (b) DBU, TU-3, DBU/TU-3 (1/1), 0.5M [DBU] ([TU-3]) in $CDCl_3$ 31

Supplementary Figure 29. (a)^{1}H NMR spectrum of the crude product of entry 3, Table 2; (b) ^{1}H NMR spectrum of the purified product of entry 3, Table 2; (c) ^{13}C NMR spectrum of the purified product of entry 3, Table 2………………………………………………………………………………….32

Supplementary Figure 30. (a)^{1}H NMR spectrum of the crude product of entry 5, Table 2; (b) ^{1}H NMR spectrum of the purified product of entry 5, Table 2; (c) ^{13}C NMR spectrum of the purified product of entry 5, Table 2... 33

Supplementary Figure 31. (a)^{1}H NMR spectrum of the crude product of entry 1, Supplementary Table 1; (b) ^{1}H NMR spectrum of the purified product of entry 1, Supplementary Table 1; (c) ^{13}C NMR spectrum of the purified product of entry 1, Supplementary Table 1……………………………………………34

Supplementary Figure 32. ((a)^{1}H NMR spectrum of the crude product of entry 3, Supplementary Table 2; (b) ^{1}H NMR spectrum of the purified product of entry 3 , Supplementary Table 2; (c) ^{13}C NMR spectrum of the purified product of entry 3, , Supplementary Table 2……………………...…....…35

Supplementary Figure 33. (a)^{1}H NMR spectrum of the crude product of entry 4, Supplementary Table 2; (b) ^{1}H NMR spectrum of the purified product of entry 4, Supplementary Table 2; (c) ^{13}C NMR spectrum of the purified product of entry 4, Supplementary Table 2……………………………………**36**
Supplementary Figure 34. ^{1}H NMR spectrum of the crude product of entry 6, Supplementary Table 2...**37**

Supplementary Figure 35. ^{1}H NMR spectrum of the crude product of entry 11, Table 1 **................... 38**

Supplementary Table 1. COS/PO copolymerization catalyzed by organic bases and TU-1/organic base pairs at 25ºC [a]

Entry	Catalyst	[PO]: [LB]: [TU-1]: [I]	TOF (h^{-1})[b]	Copolymer selectivity [c]	Alternating degree (%)[d]	T-H linkage content (%)[e]	O/S ER product [e]	M_n (kg/mol)[f]	PDI [f]
1[g]	DBU:TU-1	250:1:1:0	9	94/6	100	>99	N.F.	21.0	1.20
2[g]	DBU:TU-1	250:1:1:1	10	95/5	100	>99	N.F.	19.0	1.13
3	**P2**	2000:1:0:1	29	99/1	100	>99	N.F.	35.6	1.15
4	**P4**:TU-1	2000:1:1:1	60	99/1	100	>99	N.F.	18.7	1.14
5	**P1**:TU-1	250:1:1:0	1	>99	100	>99	N.F.	8.3	1.05
6	**P1**	500:1:0:1	2	>99	100	>99	N.F.	3.7	1.10
7	**P1**:TU-1	500:1:1:1	3	>99	100	>99	N.F.	3.5	1.12
8	MTBD	500:1:0:1	7	95/5	100	>99	N.F.	8.5	1.14
9	MTBD:TU-1	500:1:1:1	10	98/2	100	>99	N.F.	19.2	1.13
10	TBD	100:1:0:0	-	0/100	-	-	-	-	-
11	TU-1	100:1:0:0	-	-	-	-	-	-	-
12	DBU	2000:1:0:0	-	-	-	-	-	-	-

[a] Reactions were run at 25ºC in neat PO (1.0 ml; COS: PO = 1.2: 1) in a 10 ml autoclave for 24 h.
[b] (Mol epoxide consumed)/(mol cat h), PO conversion was determined by ^{1}H NMR spectroscopy.
[c] Determined by ^{1}H NMR spectroscopy. The polymer selectivity is the molar ratio of the copolymer/cyclic product.
[d] Determined by ^{1}H NMR spectroscopy. The PPMTC linkages are the molar percentage of monothiocarbonate linkage in polymer chain.
[e] Determined by ^{13}C NMR spectroscopy. O/S ER = oxygen-sulfur exchange reaction. N.F. = not found.
[f] Determined by gel permeation chromatography in THF, calibrated with polystyrene standards.
[g] COS: PO = 1.05: 1.
Representative NMR spectra are in the supporting information part (Supplementary Figure 31).

Supplementary Table 2. COS/PO copolymerization catalyzed by TU-1/DBU at different temperature [a]

Entry	T (°C)	TOF (h^{-1})[b]	Copolymer selectivity [c]	Alternating degree (%)[d]	T-H linkage content (%)[e]	O/S ER product [e]	M_n (kg/mol)[f]	PDI [f]
1	25	9	96/4	100	>99	N.F.	42.9	1.18
2	35	12	89/11	100	>99	N.F.	32.4	1.32
3	45	16	78/22	100	>99	N.F.	35.7	1.48
4	55	16	29/71	-	-	F.	16.5	1.68
5 g	55	15	24/76	-	-	F.	17.3	1.78
6	65	8	-[h]	-	-	F.	-	-

[a] Reactions were run in neat PO (1ml; COS: PO=1.2: 1; catalyst = DBU: TU-1, PO: catalyst =500: 1: 1) in a 10 ml autoclave for 24 h.
[b] (Mol epoxide consumed)/(mol LB h).
[c] Determined by ^{1}H NMR spectroscopy. The polymer selectivity is the molar ratio of the copolymer/cyclic product.
[d] Determined by ^{1}H NMR spectroscopy. The PPMTC linkages are the molar percentage of monothiocarbonate linkage in polymer chain.
[e] Determined by ^{13}C NMR spectroscopy. O/S ER = oxygen-sulfur exchange reaction. N.F. = not found and F. = found.
[f] Determined by gel permeation chromatography in THF, calibrated with polystyrene standards.
[g] Catalyst = DBU, PO: catalyst = 500: 1.
[h] Only cycle thiocarbonates were formed. (monothiocarbonate: dithiocarbonate =92: 8)
Representative NMR spectra are in the supporting information part (Supplementary Figures 32~34).

Supplementary Table 3. Copolymerization of COS with PO in the presence of different amounts of BnOH.[a]

Entry	[PO]: [BnOH]	PO conv. (%)[b]	Copolymer selec. (%)[b]	M_n^{Theo} (kg/mol)[c]	M_n^{NMR} (kg/mol)[d]	M_n^{GPC} (kg/mol)[e]	PDI [e]
1	250:1	79	98	23.4	-	18.0	1.19
2	250:2	80	98	11.9	9.0	10.0	1.19
3	250:3	79	97	7.9	6.5	7.0	1.18
4	250:4	80	98	6.0	5.3	5.7	1.17
5	250:5	82	95	4.9	4.8	4.5	1.17

[a] Copolymerizations were performed in a 10 mL autoclave at 25 °C for 24 h, [DBU]/[TU-1]/[PO] = 1/1/250, [COS]/[PO] = 1.2/1 in neat PO.

[b] Determined by the integration of ^{1}H NMR signals. The copolymer selectivity is the molar ratio of the copolymer/cyclic product.

[c] $M_n^{theo} = M_{BnOH} + 118.15 \times \frac{[PO]}{[BnOH]} \times PO\ conv.$

[d] $M_n^{NMR} = \frac{A_{5.16}}{A_{4.01}} \times 118.15 + M_{BnOH}$, calculated from the ^{1}H NMR spectroscopy of the copolymers (Supplementary Figure 16).

[e] GPC in THF at 40 °C, polystyrene standard (Supplementary Figure 17).

Supplementary Table 4. Copolymerization of COS with PO under various [PO]/[H_2O] ratios.^{a}

entry	[PO]/[H_2O]	PO conv.(%)[b]	Copolym. Selec. (%)[b]	M_n^{Theo} (kg/mol) [c]	M_n^{NMR} (kg/mol)[d]	M_n^{GPC} (kg/mol)[e]	PDI[e]
1	100:2	68	91	4.0	3.8	4.1	1.11
2	100:3	65	90	2.6	2.2	2.4	1.10
3	100:4	64	92	1.9	1.5	1.7	1.13
4	100:5	63	93	1.5	1.3	1.2	1.14

[a] Copolymerizations were performed in a 10 mL autoclave at 25 °C for 24 h, [DBU]/[TU-1]/[PO] = 1/1/250, [COS]/[PO] = 1.2/1 in neat PO.

[b] Determined by the integration of ^{1}H NMR signals. The copolymer selectivity is the molar ratio of the copolymer/cyclic product.

[c] $M_n^{theo} = M_{H2O} + 118.15 \times \frac{[PO]}{[H2O]} \times PO\ conv.$

[d] $M_n^{NMR} = \frac{A_{5.16}}{A_{4.01}} \times 118.15 \times 2$, calculated based on the ^{1}H NMR spectroscopy of the copolymers (Supplementary Figure 19）

[e] GPC in THF at 40 °C, polystyrene standard (Supplementary Figure 20).

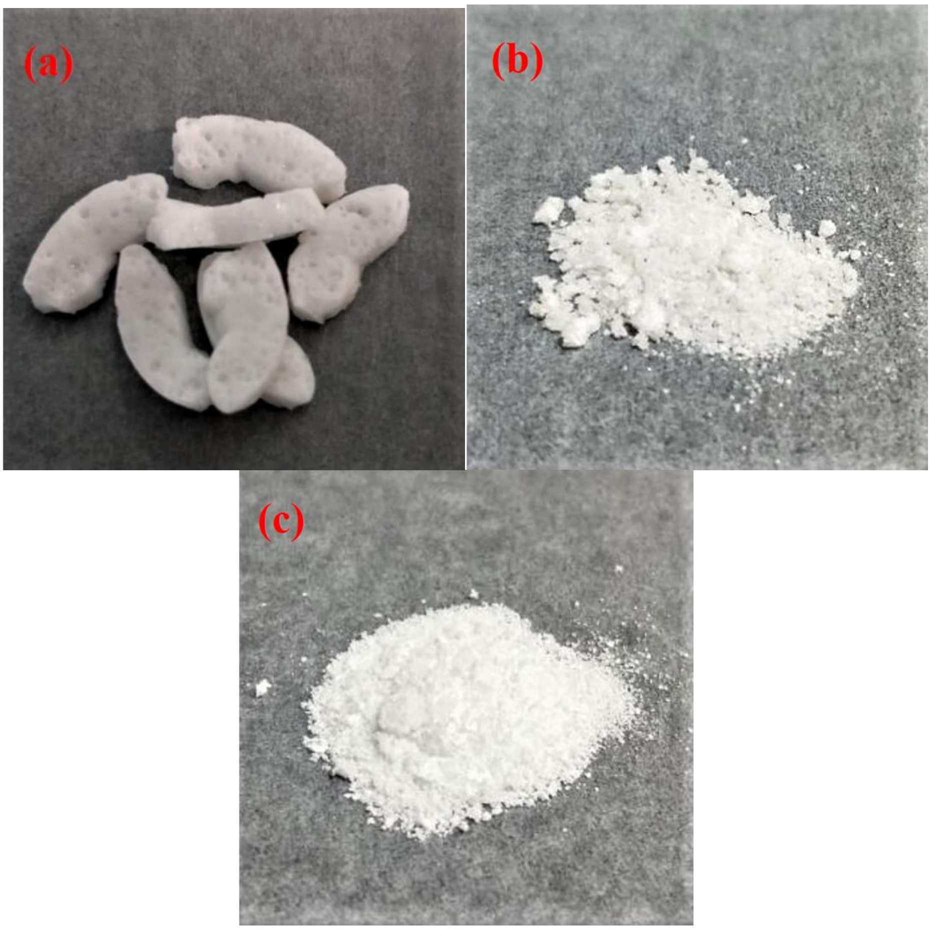

Supplementary Figure 1. The photos of (a) PO/COS, (b) PGE/COS, (c) CHO/COS copolymers.

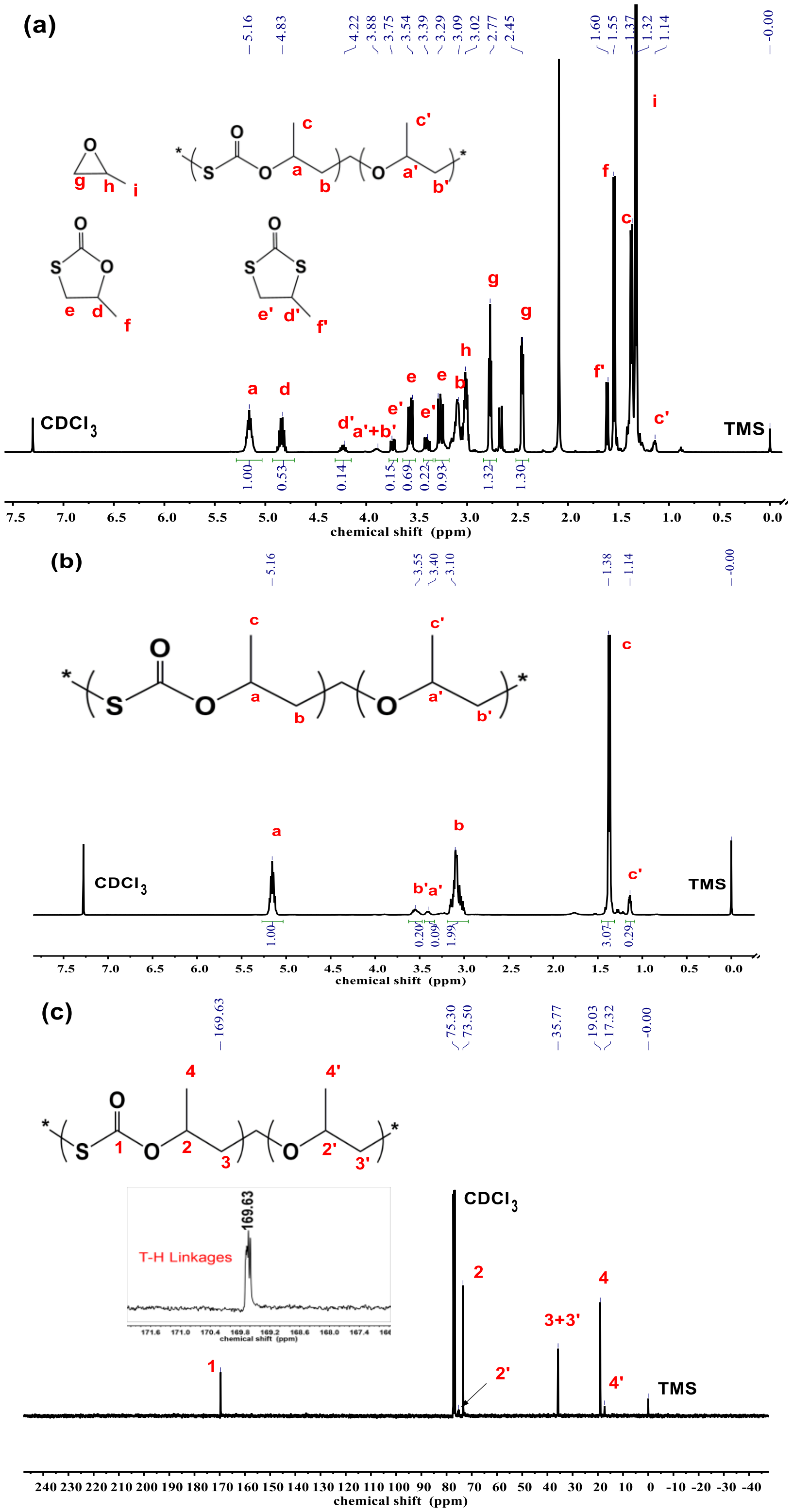

Supplementary Figure 2. (a)^{1}H NMR spectrum of the crude product of entry 2, Table 1; (b) ^{1}H NMR spectrum of the purified product of entry 2, Table 1; (c) ^{13}C NMR spectrum of the purified product of entry 2, Table 1.

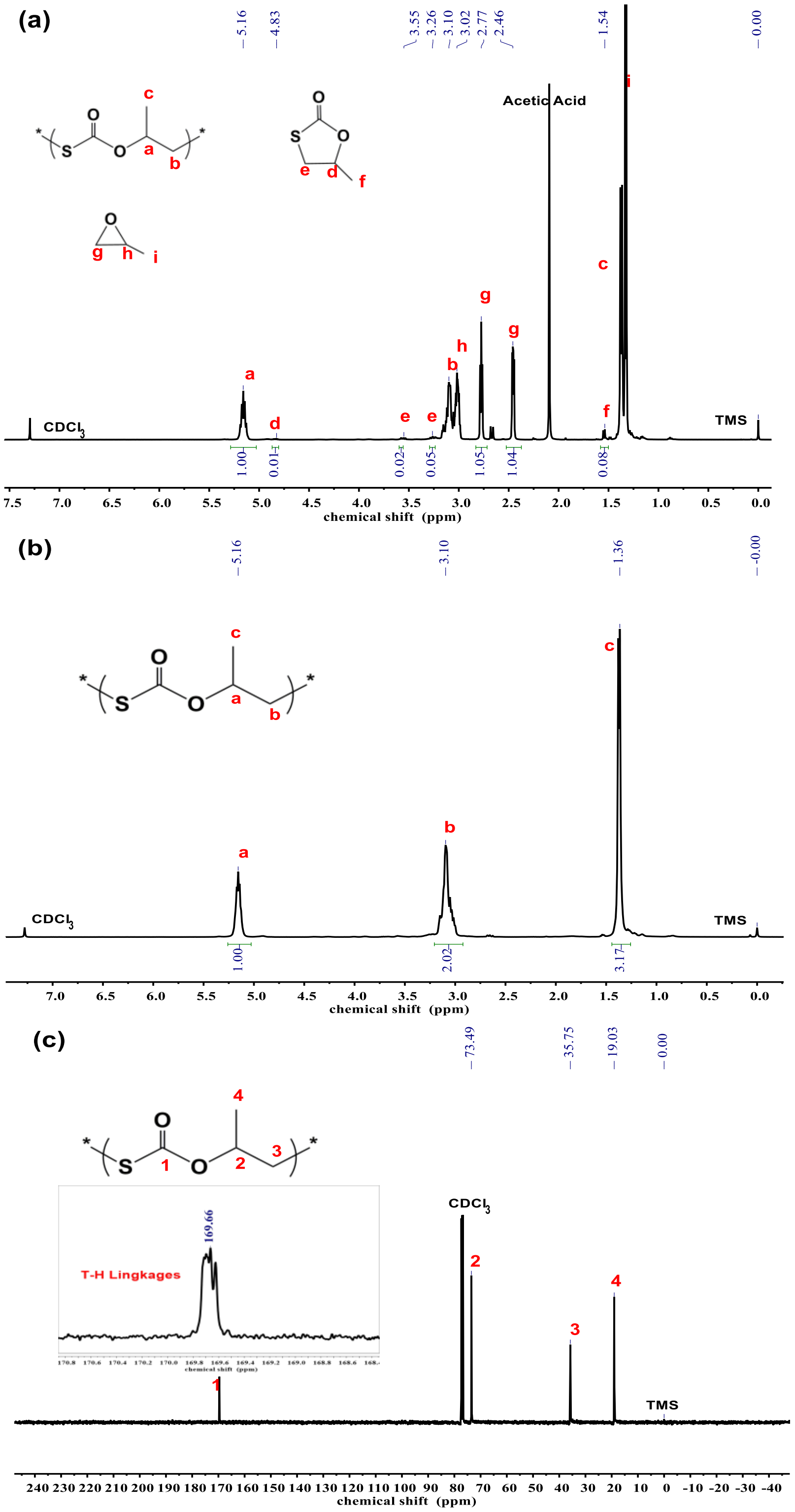

Supplementary Figure 3. (a)^{1}H NMR spectrum of the crude product of entry 9, Table 1; (b) ^{1}H NMR spectrum of the purified product of entry 9, Table 1; (c) ^{13}C NMR spectrum of the purified product of entry 9, Table 1.

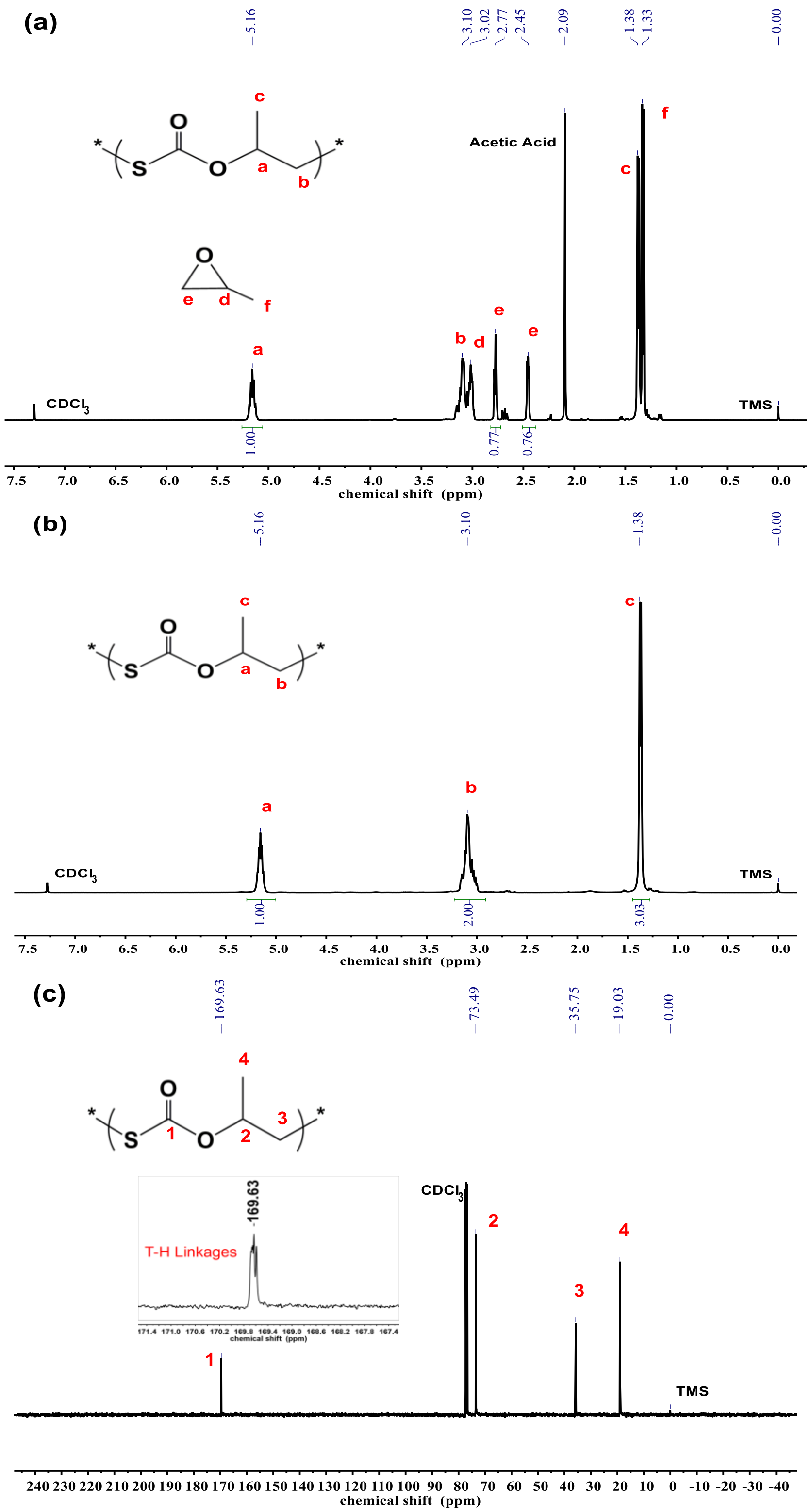

Supplementary Figure 4. (a)^{1}H NMR spectrum of the crude product of entry 10, Table 1; (b) ^{1}H NMR spectrum of the purified product of entry 10, Table 1; (c) ^{13}C NMR spectrum of the purified product of entry 10, Table 1.

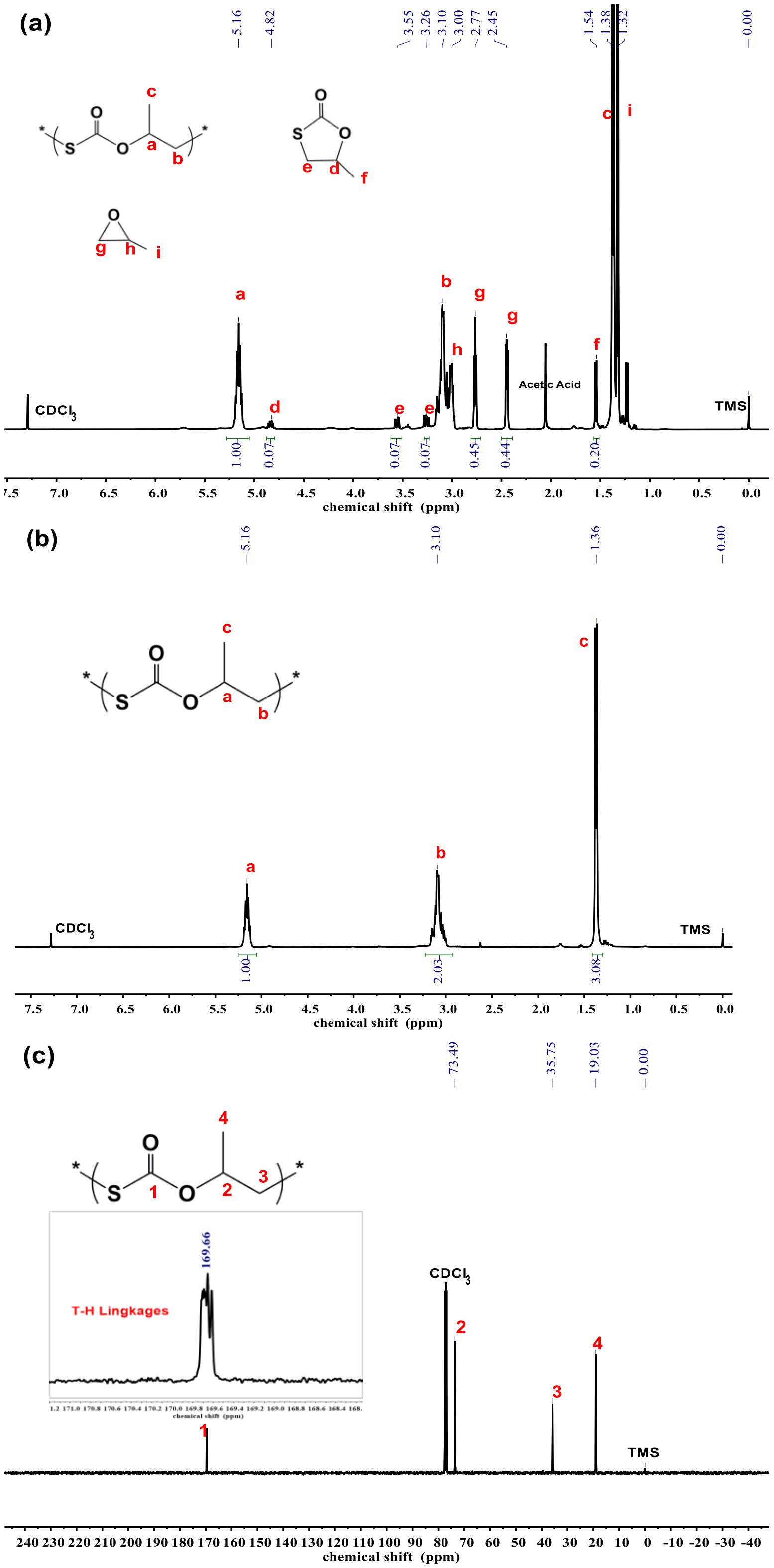

Supplementary Figure 5. (a)^{1}H NMR spectrum of the crude product of entry 11, Table 1; (b) ^{1}H NMR spectrum of the purified product of entry 11, Table 1; (c) ^{13}C NMR spectrum of the purified product of entry 11, Table 1.

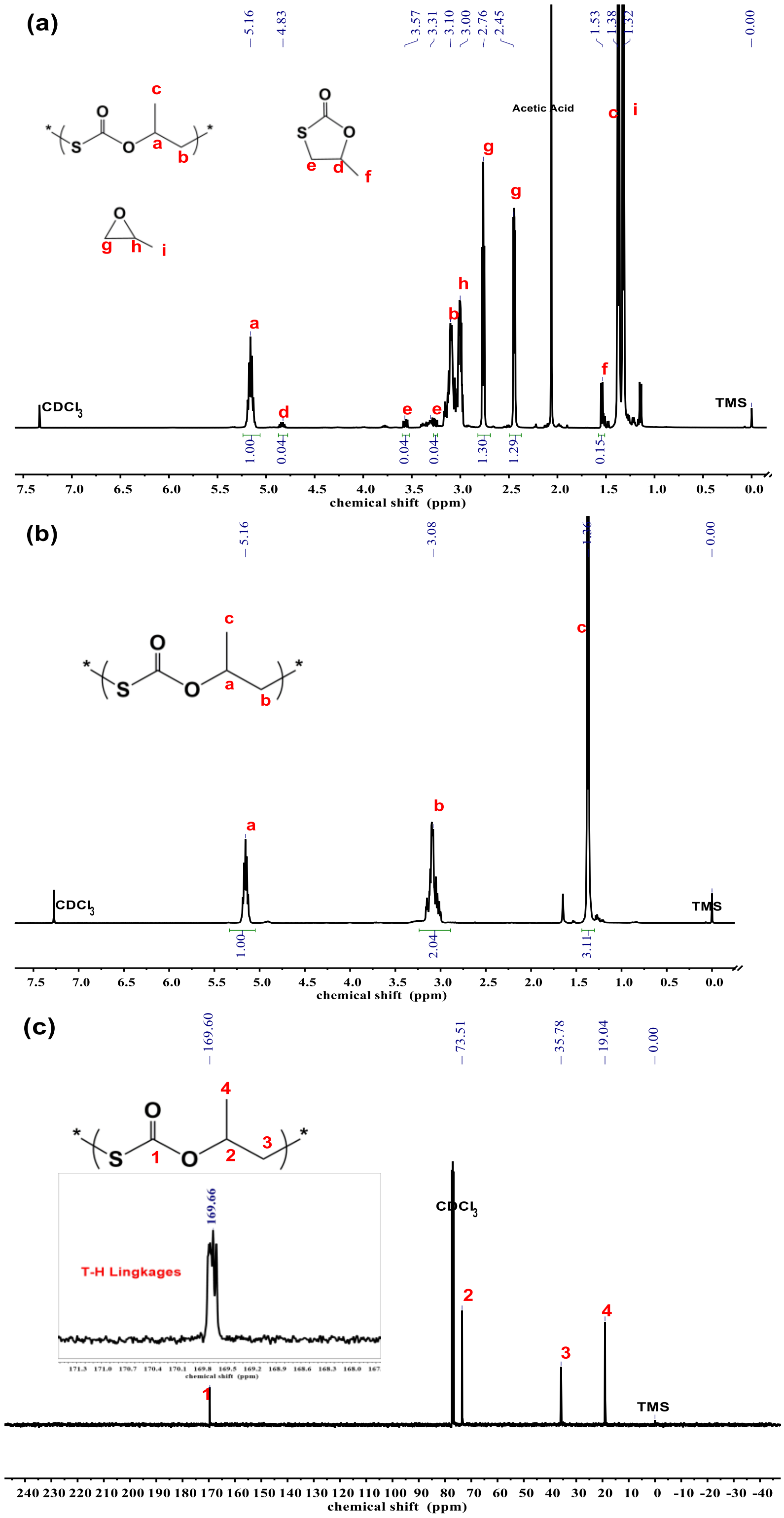

Supplementary Figure 6. (a)^{1}H NMR spectrum of the crude product of entry 12, Table 1; (b) ^{1}H NMR spectrum of the purified product of entry 12, Table 1; (c) ^{13}C NMR spectrum of the purified product of entry 12, Table 1.

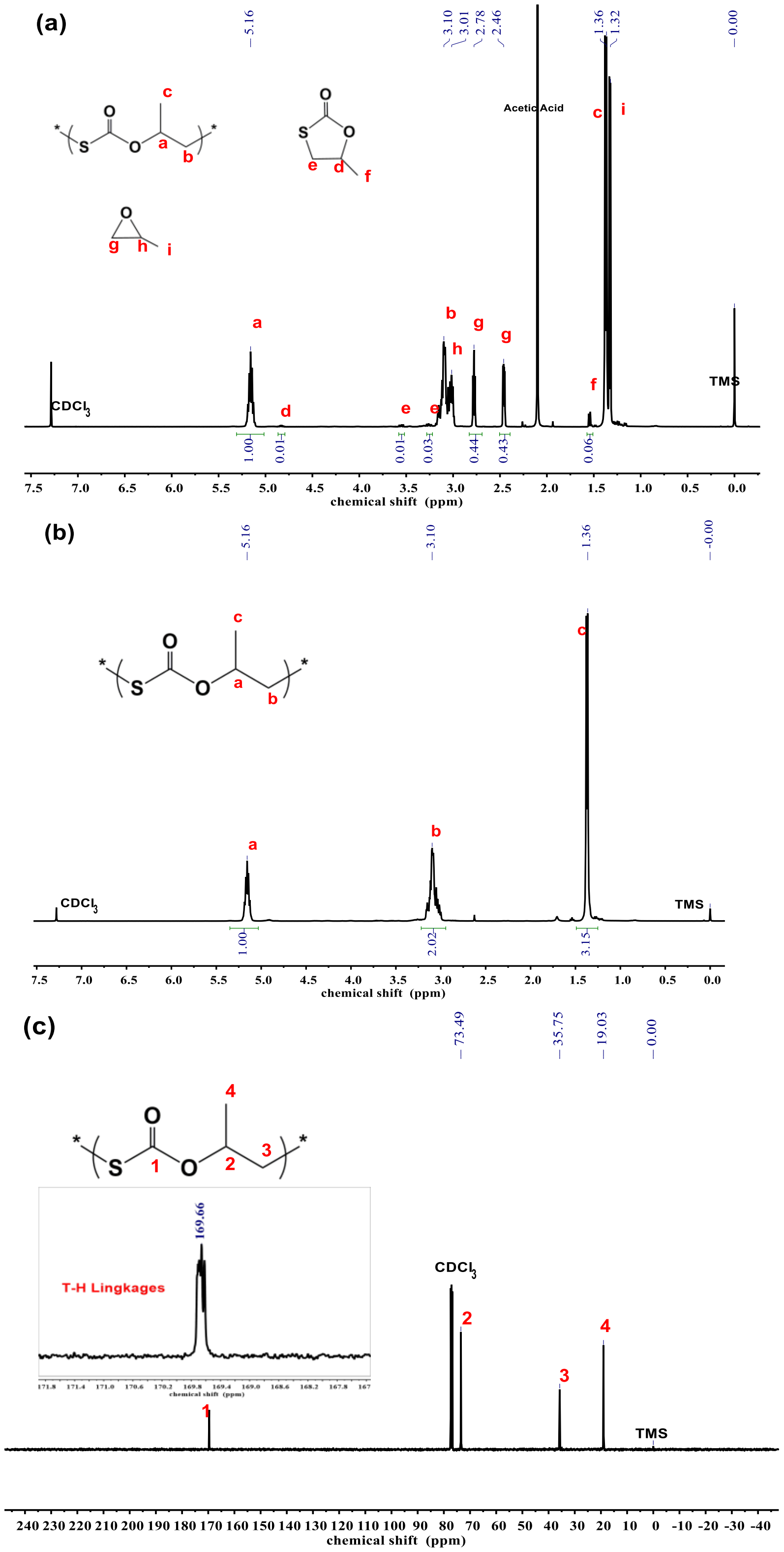

Supplementary Figure 7. (a)^{1}H NMR spectrum of the crude product of entry 13, Table 1; (b) ^{1}H NMR spectrum of the purified product of entry 13, Table 1; (c) ^{13}C NMR spectrum of the purified product of entry 13, Table 1.

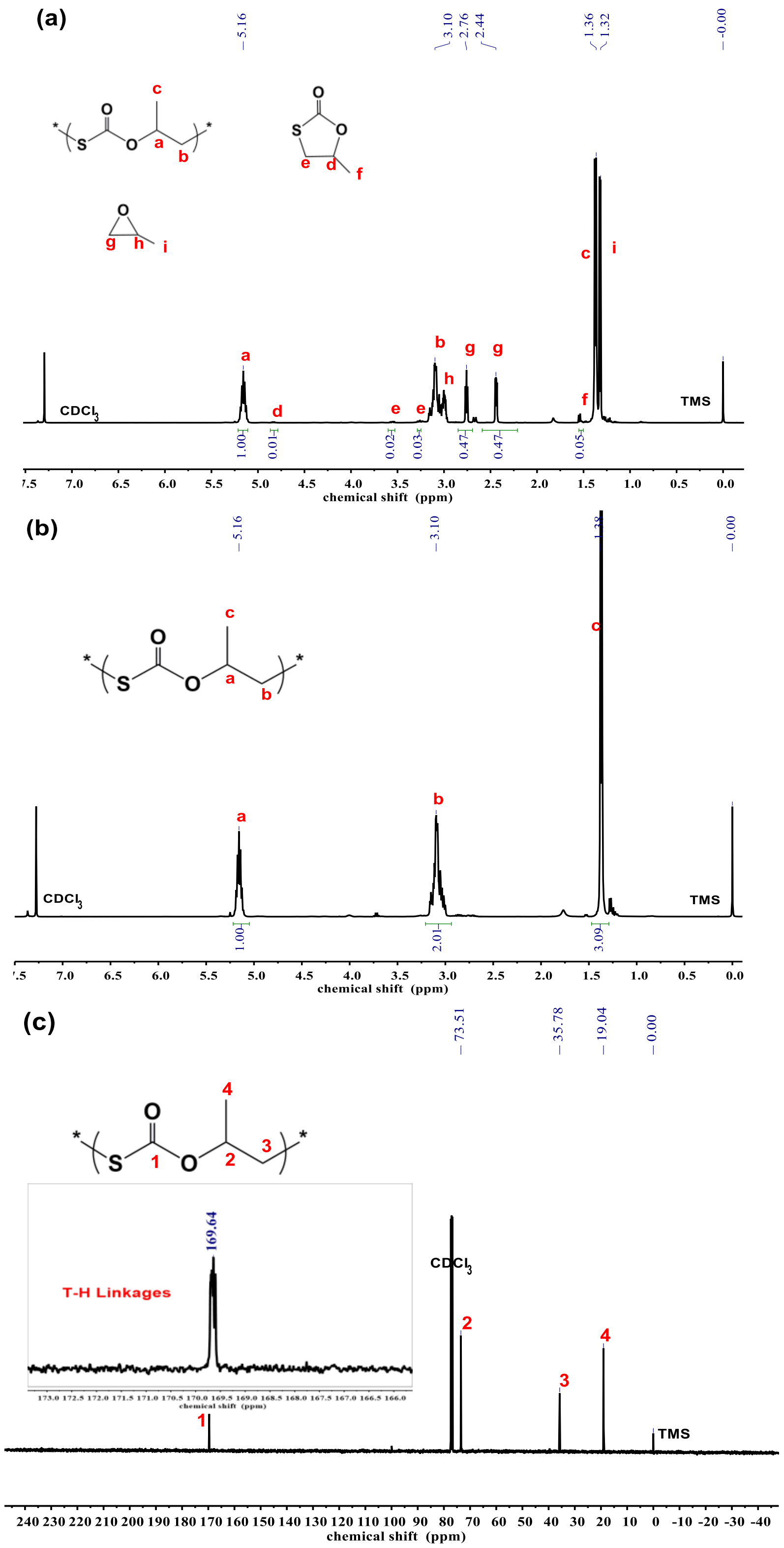

Supplementary Figure 8. (a)^{1}H NMR spectrum of the crude product of entry 15, Table 1; (b) ^{1}H NMR spectrum of the purified product of entry 15, Table 1; (c) ^{13}C NMR spectrum of the purified product of entry 15, Table 1.

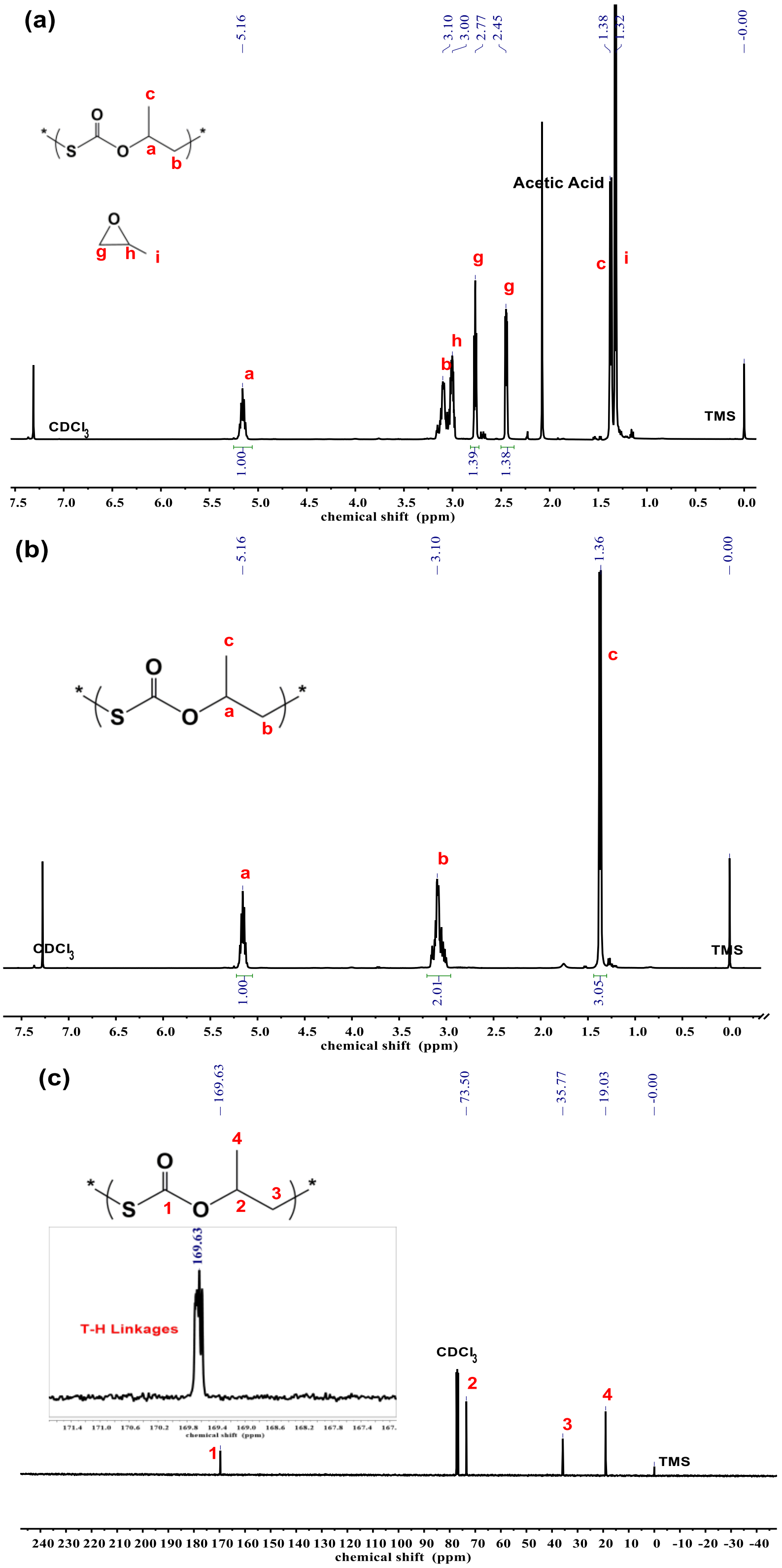

Supplementary Figure 9. (a)^{1}H NMR spectrum of the crude product of entry 16, Table 1; (b) ^{1}H NMR spectrum of the purified product of entry 16, Table 1; (c) ^{13}C NMR spectrum of the purified product of entry 16, Table 1.

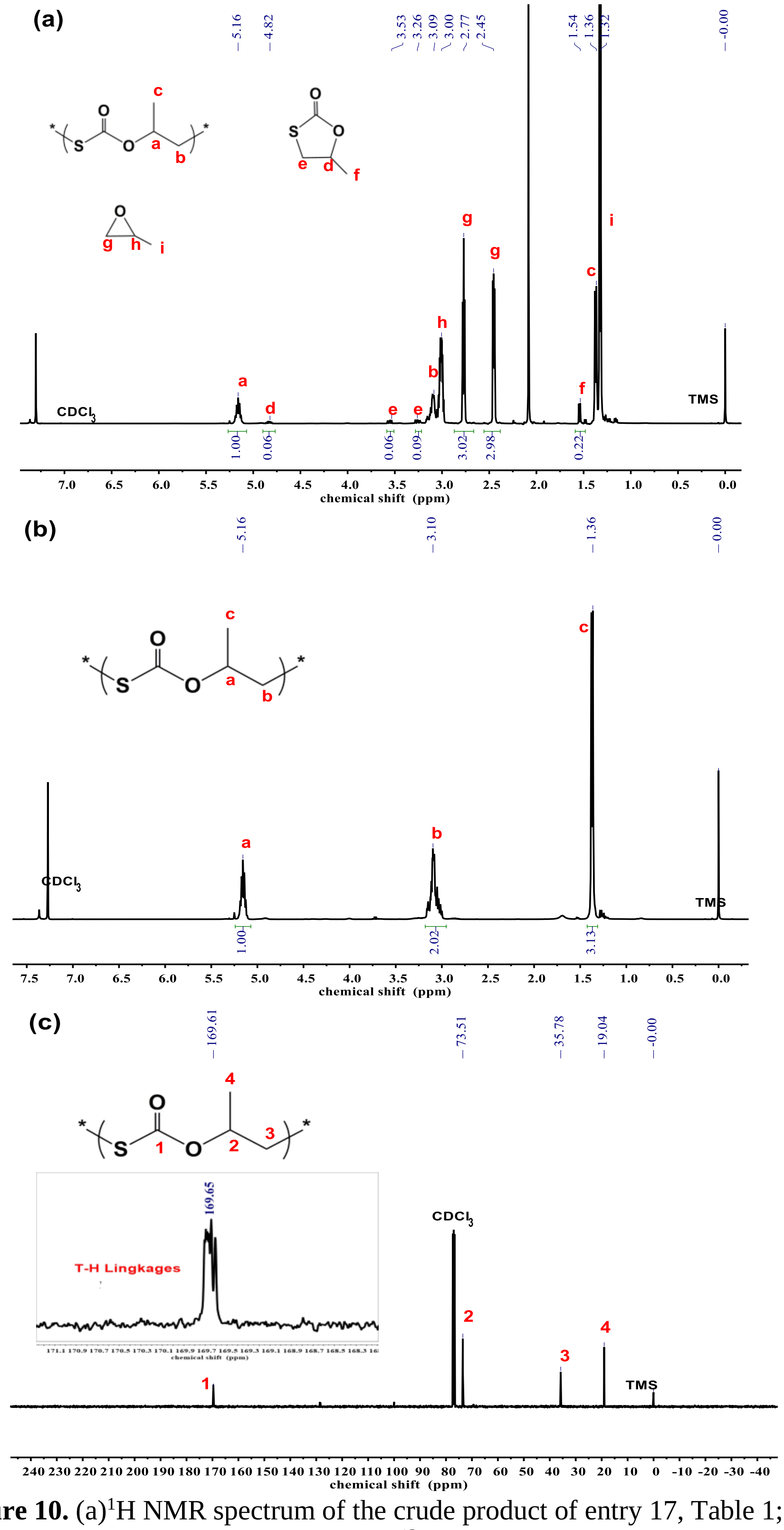

Supplementary Figure 10. (a) ^{1}H NMR spectrum of the crude product of entry 17, Table 1; (b) ^{1}H NMR spectrum of the purified product of entry 17, Table 1; (c) ^{13}C NMR spectrum of the purified product of entry 17, Table 1.

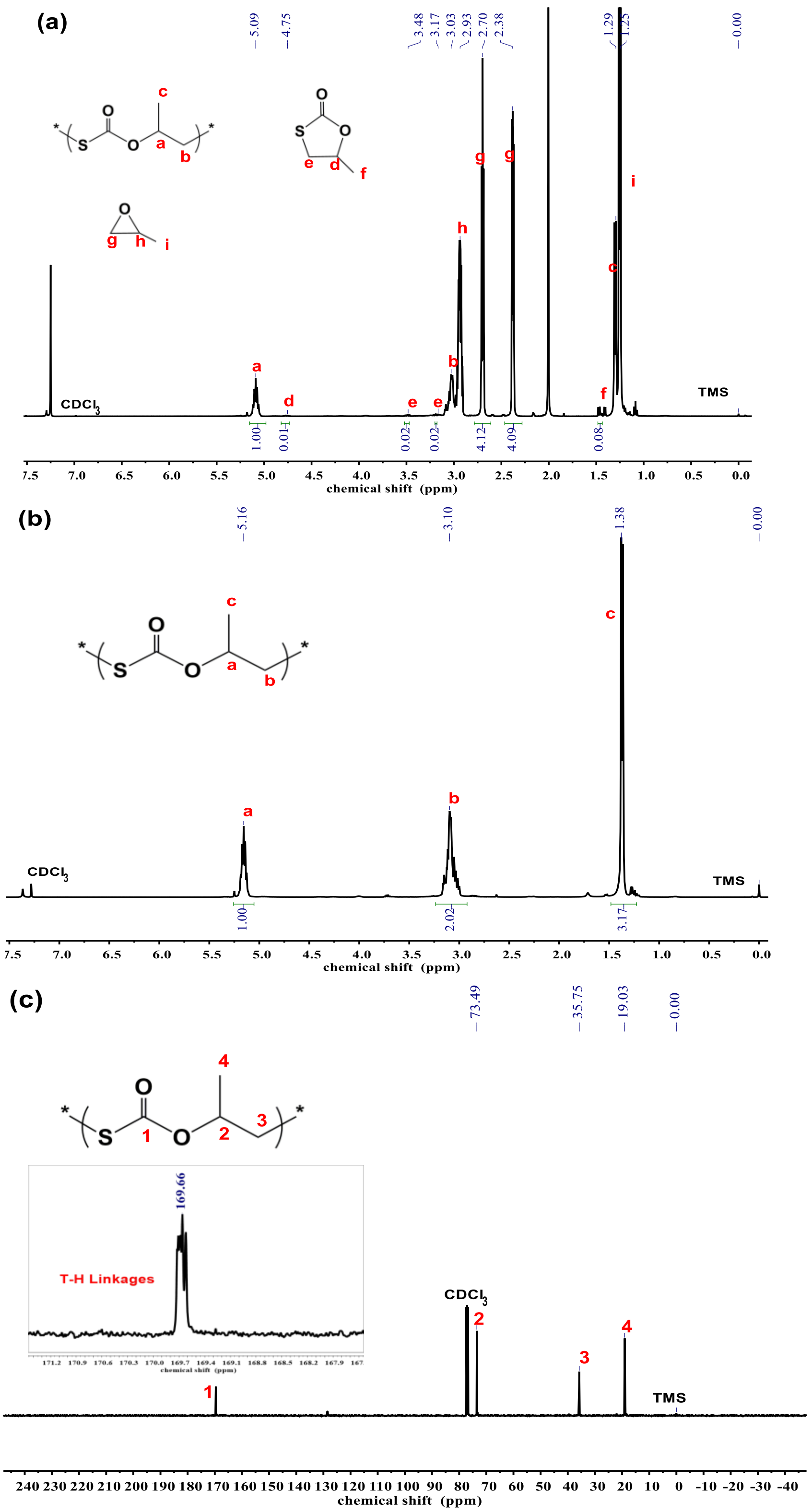

Supplementary Figure 11. (a)^{1}H NMR spectrum of the crude product of entry 18, Table 1; (b) ^{1}H NMR spectrum of the purified product of entry 18, Table 1; (c) ^{13}C NMR spectrum of the purified product of entry 18, Table 1.

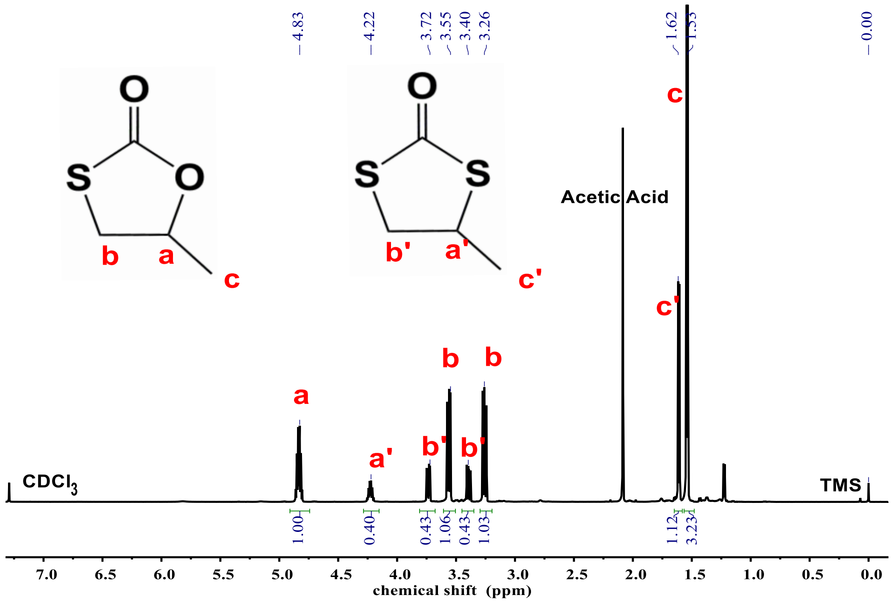

Supplementary Figure 12. ^{1}H NMR spectra of crude products in the control experiment. Reactions were run at 65 °C in neat PO (1.0 ml; COS:PO = 1.2:1; catalyst = DBU:TU-1, PO: catalyst = 500:1) in a 10 ml autoclave for 12 h. After that, two kinds of cyclic thiocarbonates were formed and PO was consumed completely, which was confirmed by ^{1}H NMR of the crude products (above). And then, the crude products which contain catalysts were restarting to stirred at 25 °C. No variation was found after 12 h from the ^{1}H NMR of the crude products.

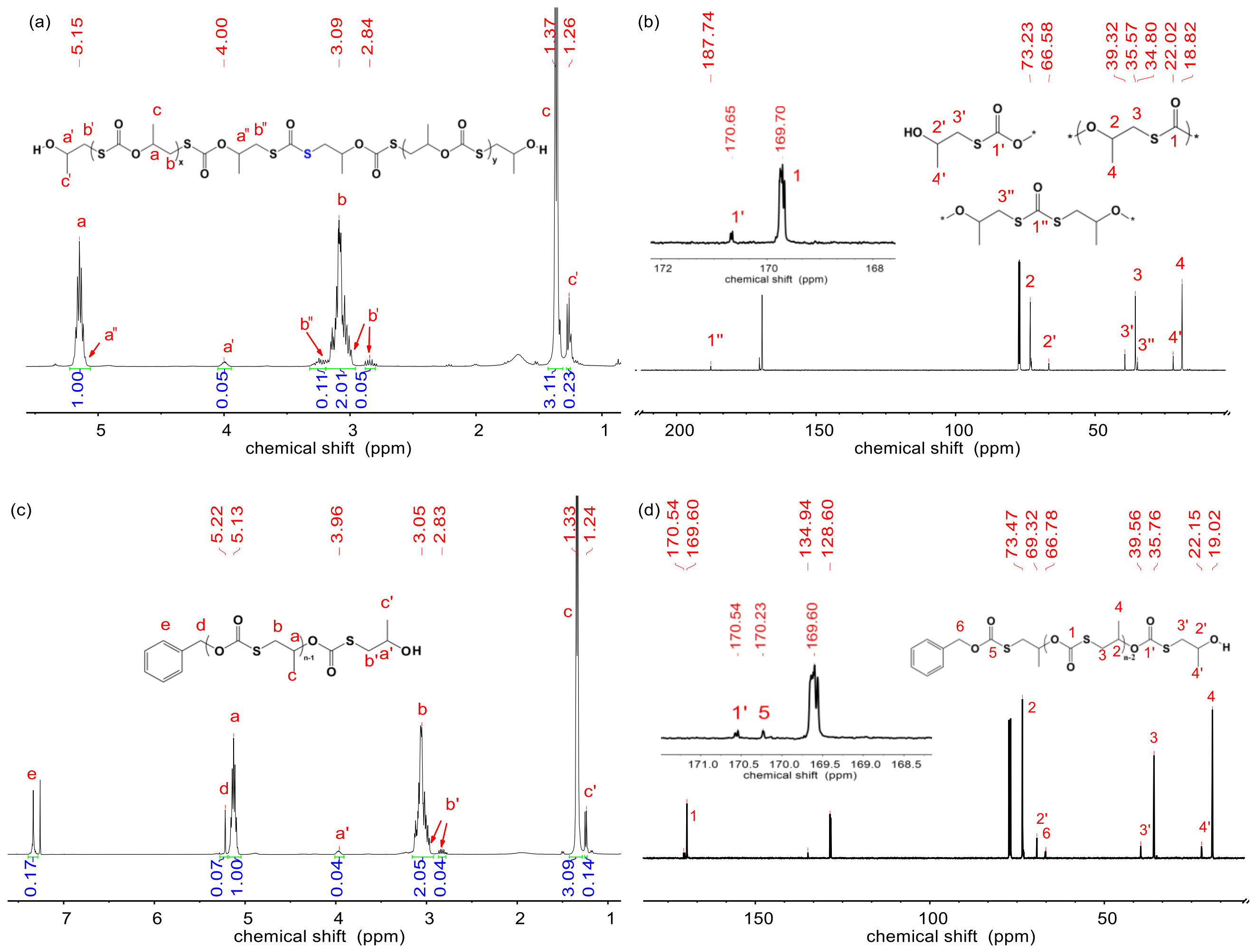

Supplementary Figure 13. PO: DBU: TU-1 of 100: 1: 1 was performed for 3.5 h at 25°C, (a) 600 MHz ^{1}H NMR spectra, (b) 125 MHz ^{13}C NMR spectra; PO: DBU: TU-1: BnOH of 100: 1: 1: 1 was performed for 3.5 h at 25°C, (c) 600 MHz ^{1}H NMR spectra, (d) 125 MHz ^{13}C NMR spectra.

Supplementary Kinetic Plots
For all plots were run at 25°C in neat PO (1.0 ml; COS: PO = 1.2: 1) in a 10 ml autoclave.

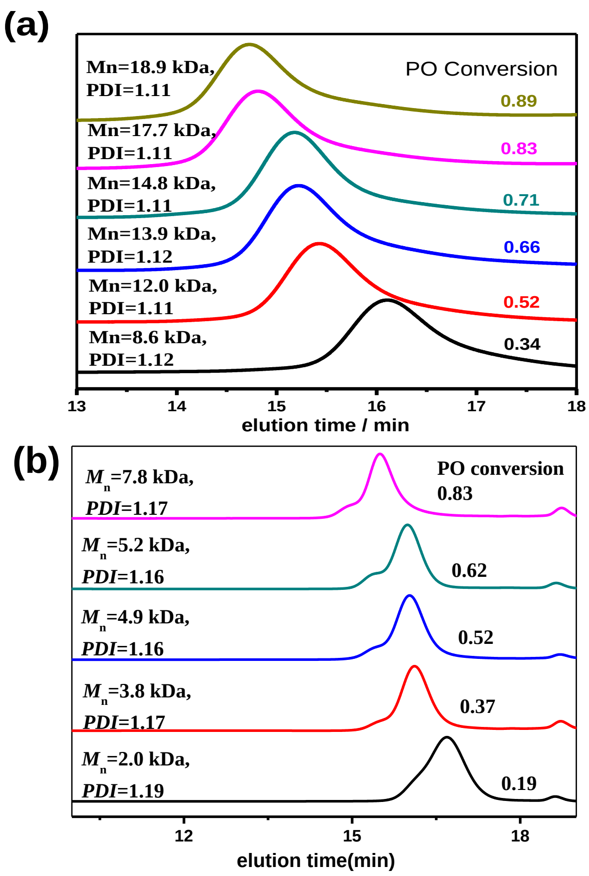

Supplementary Figure 14. (a) GPC curves when [DBU] = [TU-1] = 0.2 M; (b) GPC curves when [DBU] = [TU-1] = 0.2 M, [BnOH] = 0.08M.

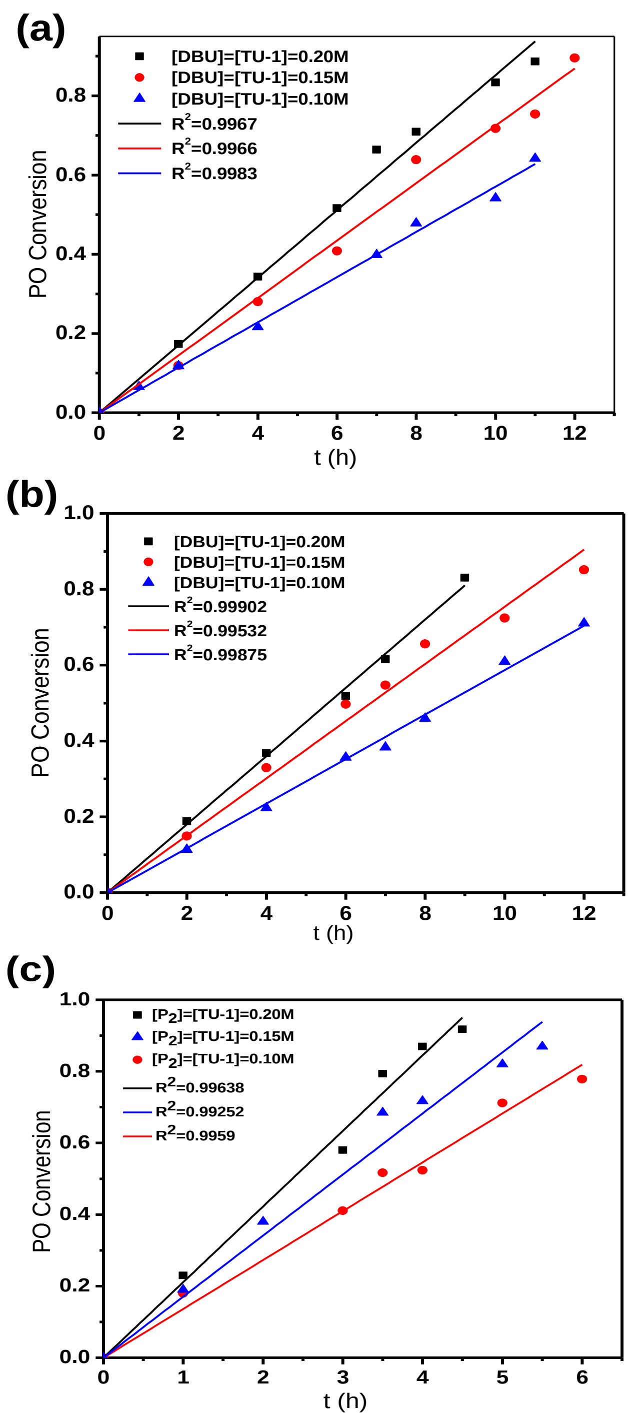

Supplementary Figure 15. (a) Zero-order kinetic plots for copolymerization of COS and PO when [DBU] = [TU-1], (b) Zero-order kinetic plots for copolymerization of COS and PO when [DBU] = [TU-1], [BnOH] = 0.08 M, (c) Zero-order kinetic plots for copolymerization of COS and PO when [**P2**] = [TU-1].

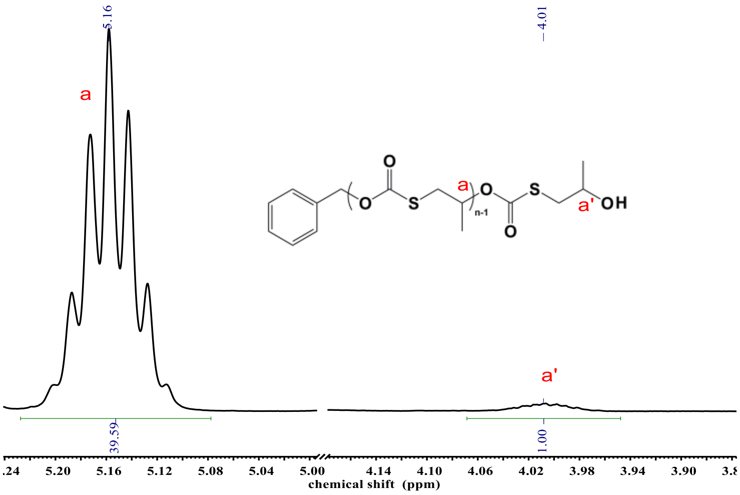

Supplementary Figure 16. ^{1}H NMR spectrum of the purified product of entry 5 in Supplementary Table 3. $M_n^{NMR} = \frac{A_{5.16}}{A_{4.01}} \times 118.15 + M_{BnOH}$,

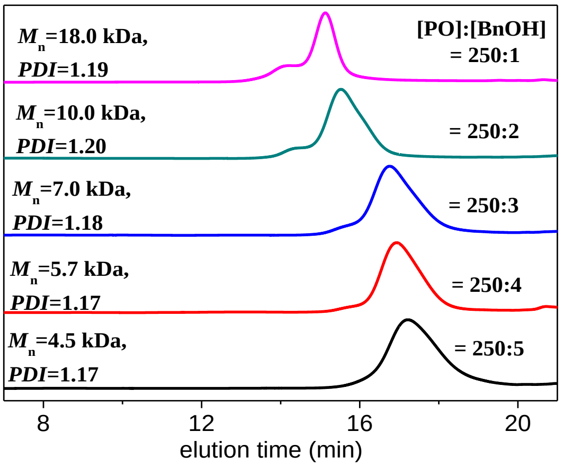

Supplementary Figure 17. GPC curves of entries 1-5 in Supplementary Table 3 (small shoulder peak at high molecular weight was caused by trace water).

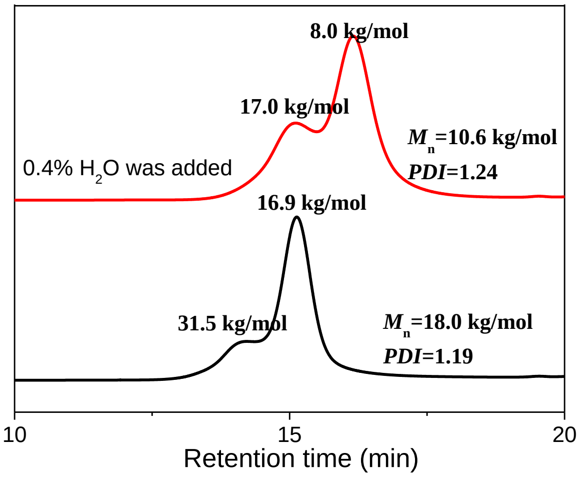

Supplementary Figure 18. GPC curves (black: entry 1 in Supplementary Table 3; red: 0.4 mol% H_2O was added under the same conditions with entry 1 in Supplementary Table 3).

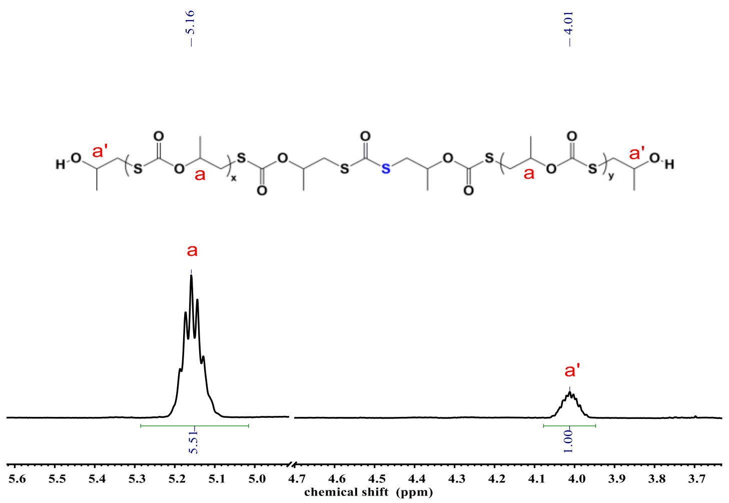

Supplementary Figure 19. ^{1}H NMR spectrum of the purified product of entry 5 in Supplementary Table 4. $M_n^{NMR} = \frac{A_{5.16}}{A_{4.01}} \times 118.15 \times 2$ (multiply by 2 means chain growth at two sides, and one chain with two -OH groups).

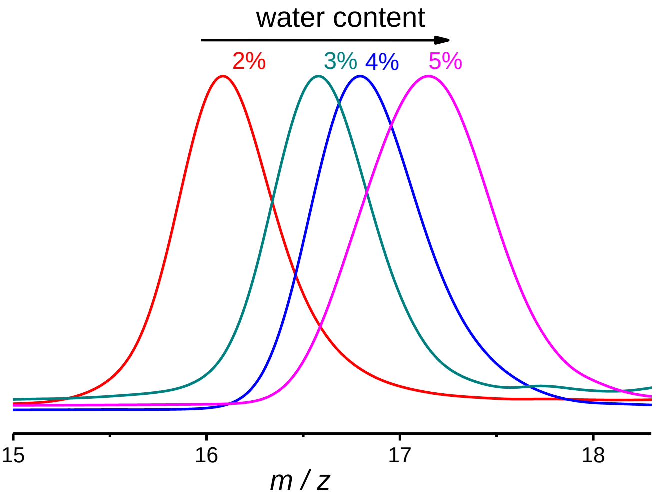

Supplementary Figure 20. GPC curves of entries 1-4 in Supplementary Table 4.

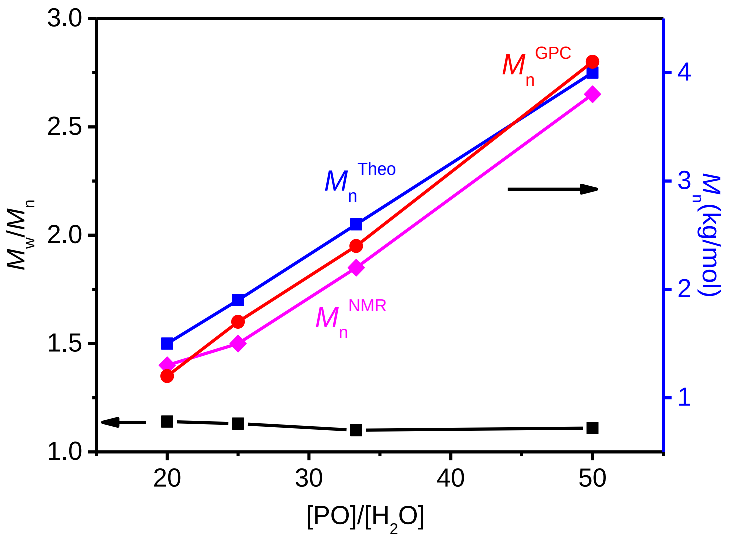

Supplementary Figure 21. The effect of H_2O content on the molecular weights of the COS/PO copolymers (Supplementary Table 4).

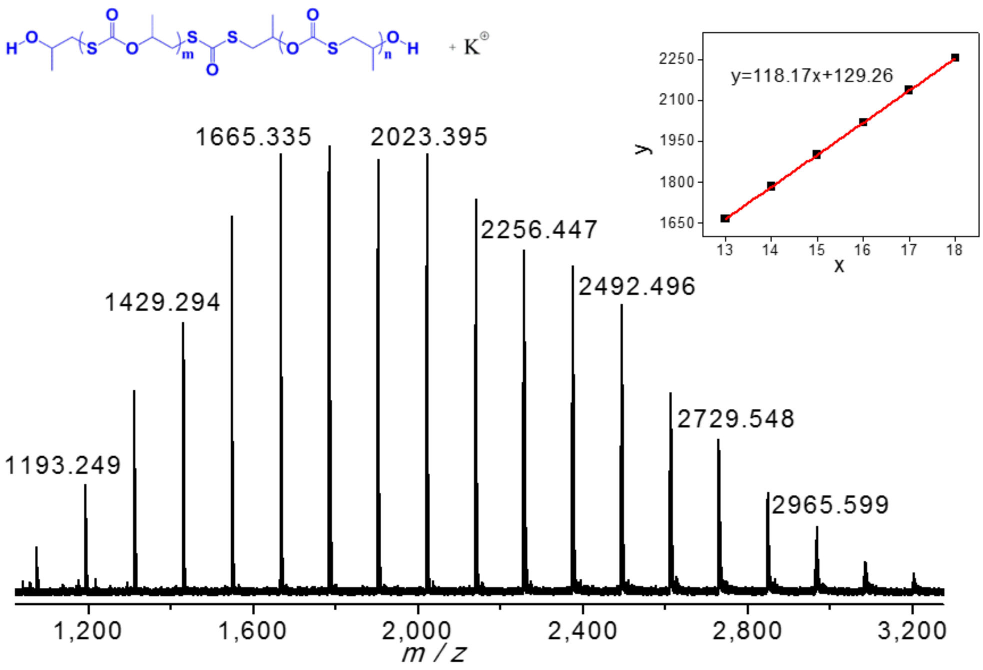

Supplementary Figure 22. MALDI-TOF MS spectra of PPTMCs in entry 3 in Supplementary Table 4.

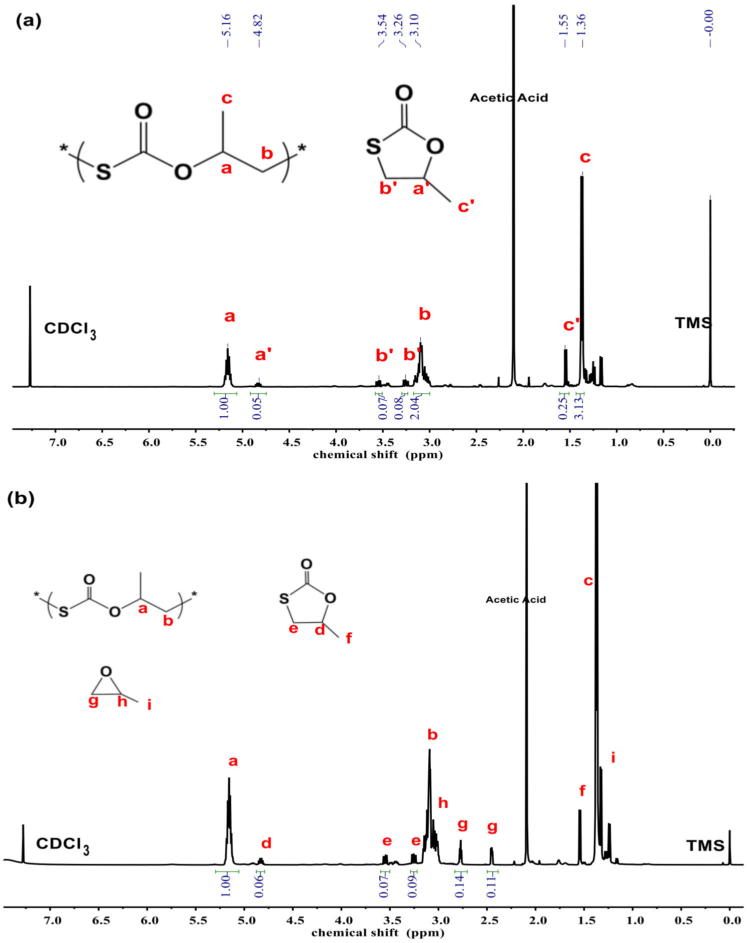

Supplementary Figure 23. Chain extension reaction. (a) PO was totally consumed according to the ^{1}H NMR spectra. (b) PO conversion was 88% according to ^{1}H NMR spectra.

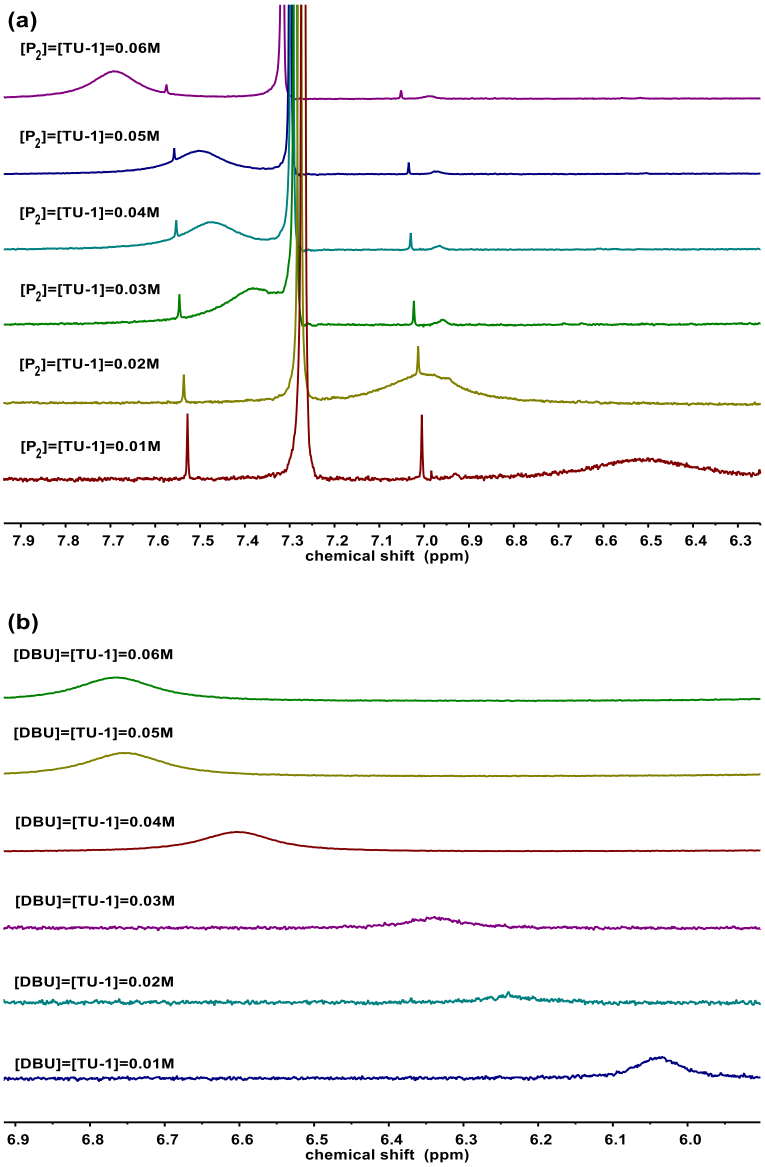

Supplementary Figure 24. ^{1}H NMR spectra of (a) the chemical shift of the *ortho*-protons of TU-1 with different concentration of [**P2**] = [TU-1] in $CDCl_3$, (b) he chemical shift of the *ortho*-protons of TU-1 with different concentration of [DBU] = [TU-1] in $CDCl_3$.

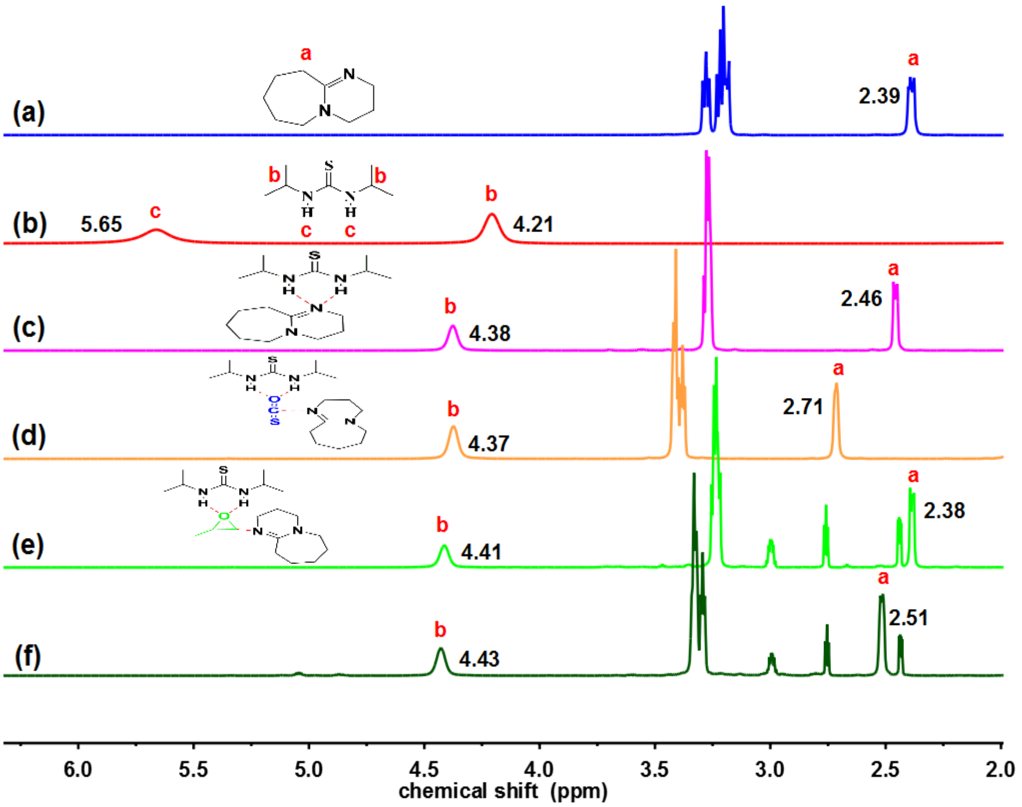

Supplementary Figure 25. ^{1}H NMR spectra of (a) DBU, (b) TU-1, (c) TU-1/DBU (1/1), (d) TU-1/DBU/COS (1/1/excess), (e) DBU/COS (1/excess), (f) TU-1/DBU/PO (1/1/1), and (g) TU-1/DBU/PO/COS (1/1/1/excess). 0.5 M TU-1 (DBU) in $CDCl_3$.

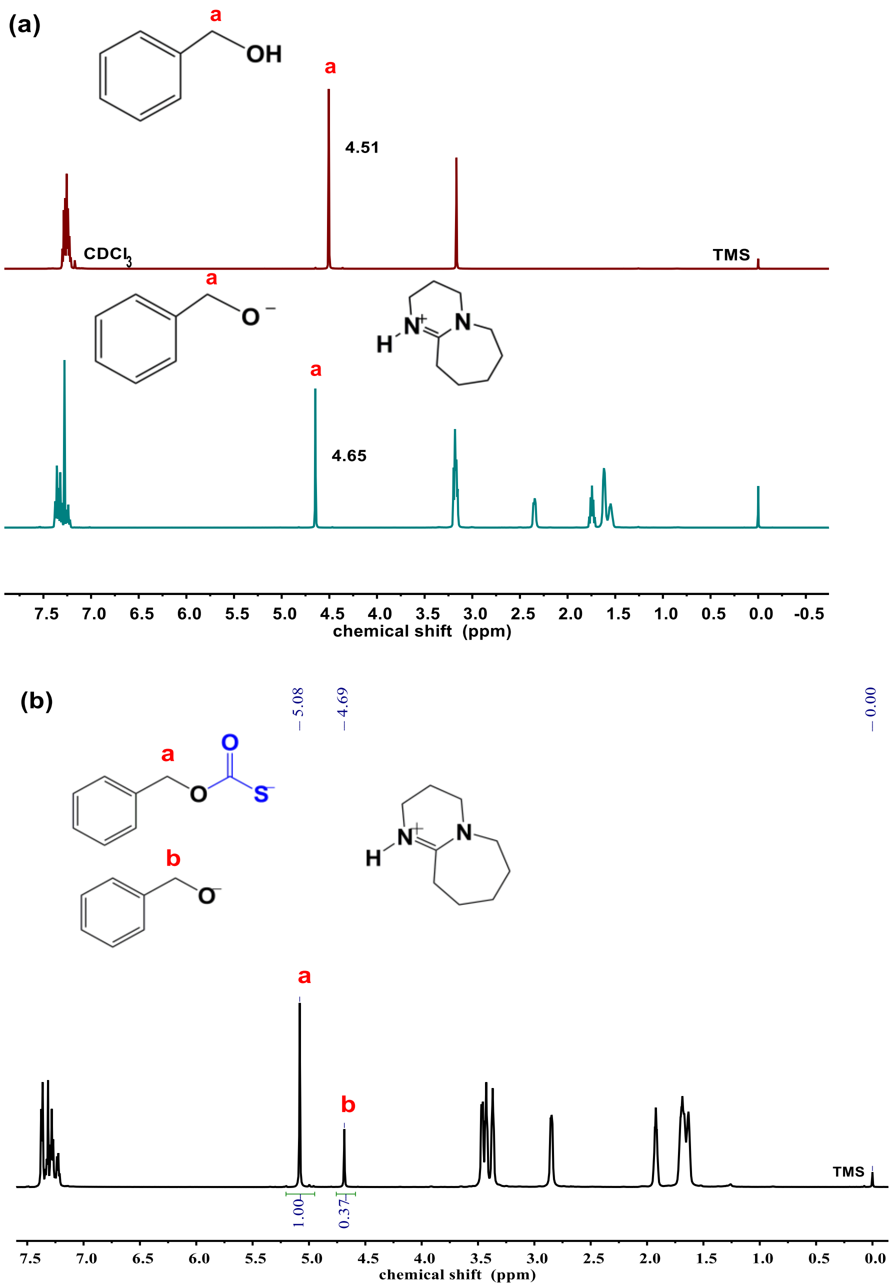

Supplementary Figure 26. ^{1}H NMR spectra of (a) BnOH, BnOH/DBU (1/1), (b) BnOH/DBU/COS (1/1/excess), 72% BnO$^-$ was equipped with COS, 0.5M [BnOH] ([DBU]) in $CDCl_3$.

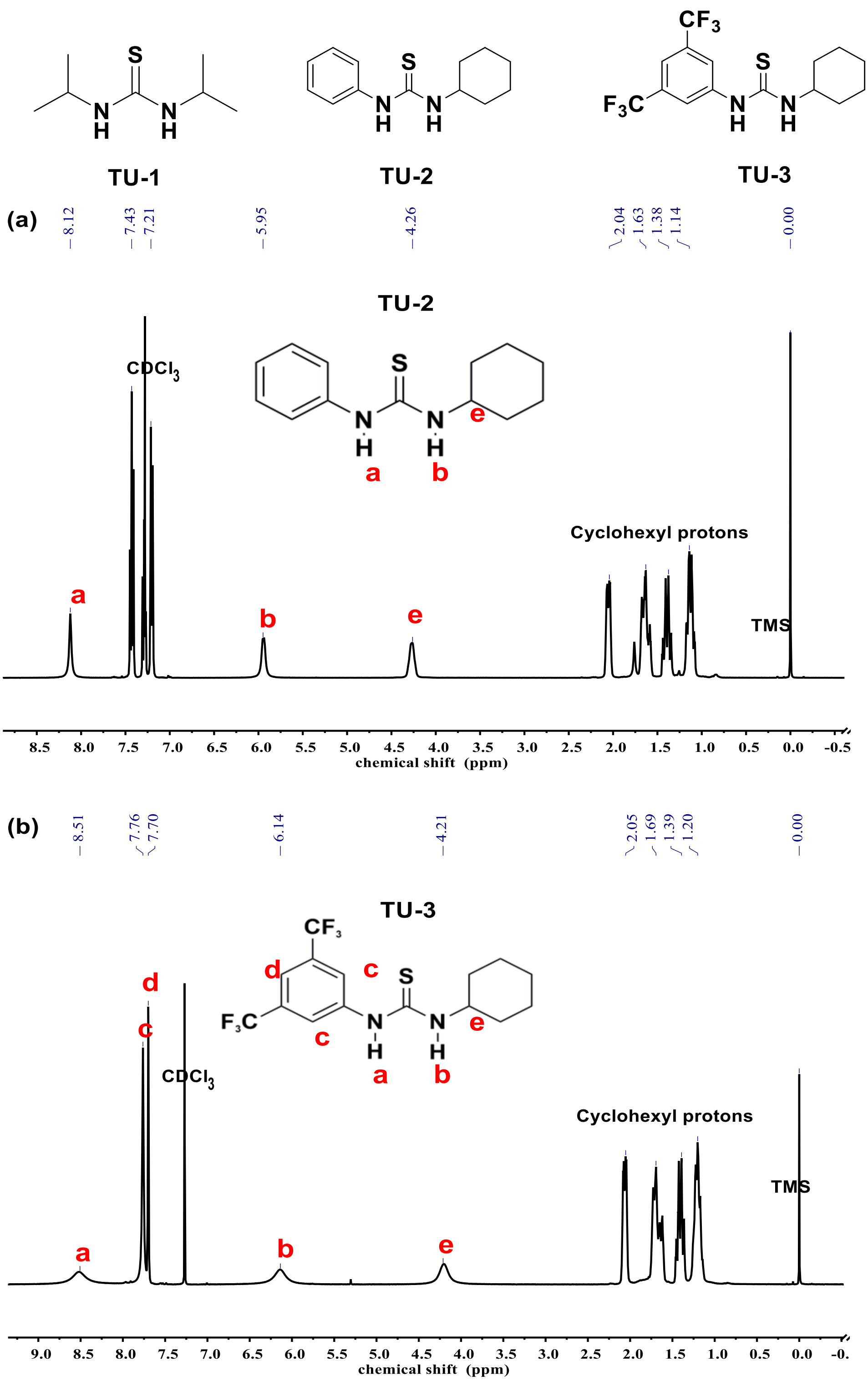

Supplementary Figure 27. ^{1}H NMR spectra of (a) TU-2, (b) TU-3 in $CDCl_3$.

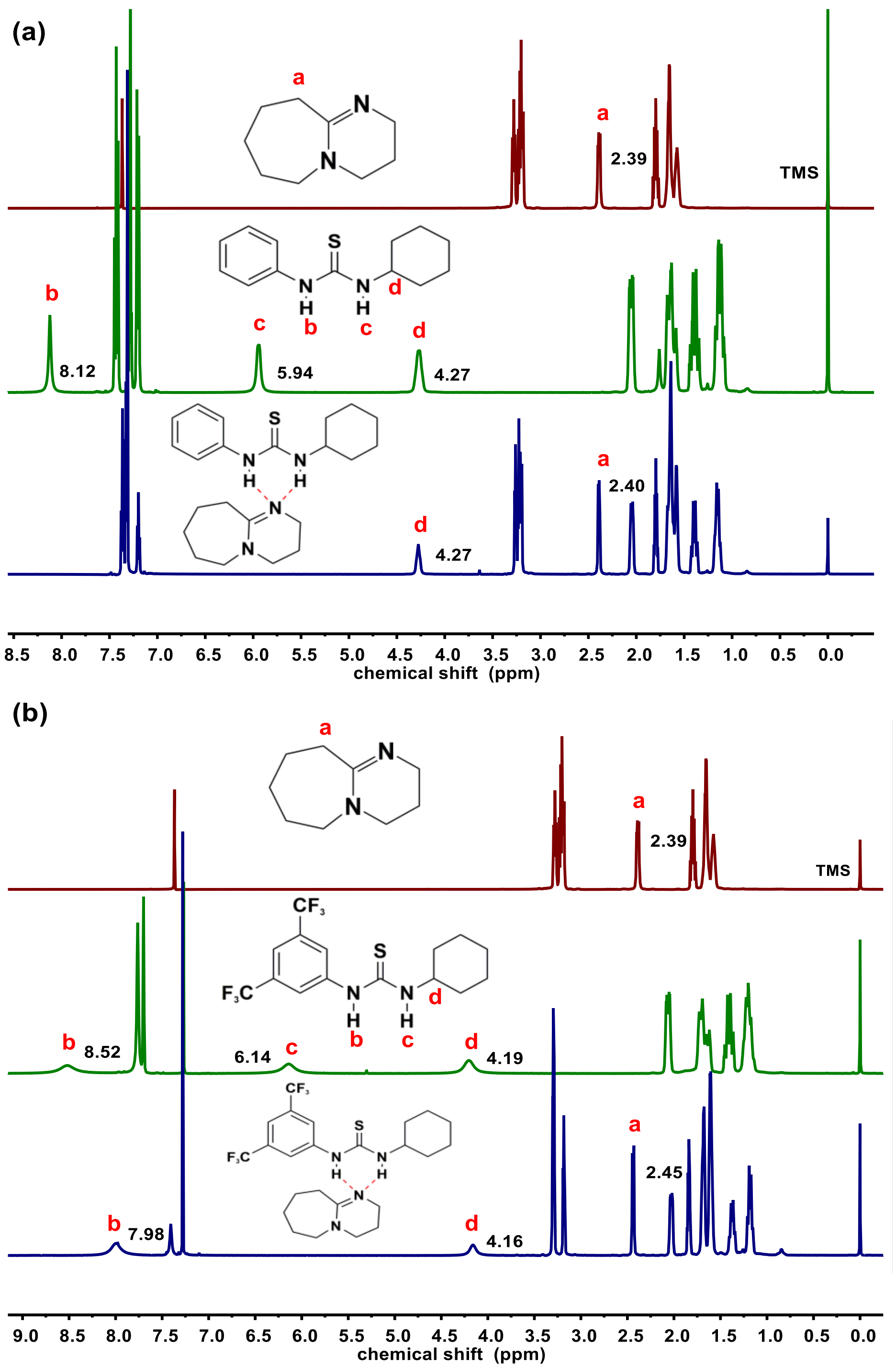

Supplementary Figure 28. ^{1}H NMR spectra of (a) DBU, TU-2, DBU/TU-2 (1/1), (b) DBU, TU-3, DBU/TU-3 (1/1), 0.5M [DBU] ([TU-3]) in $CDCl_3$.

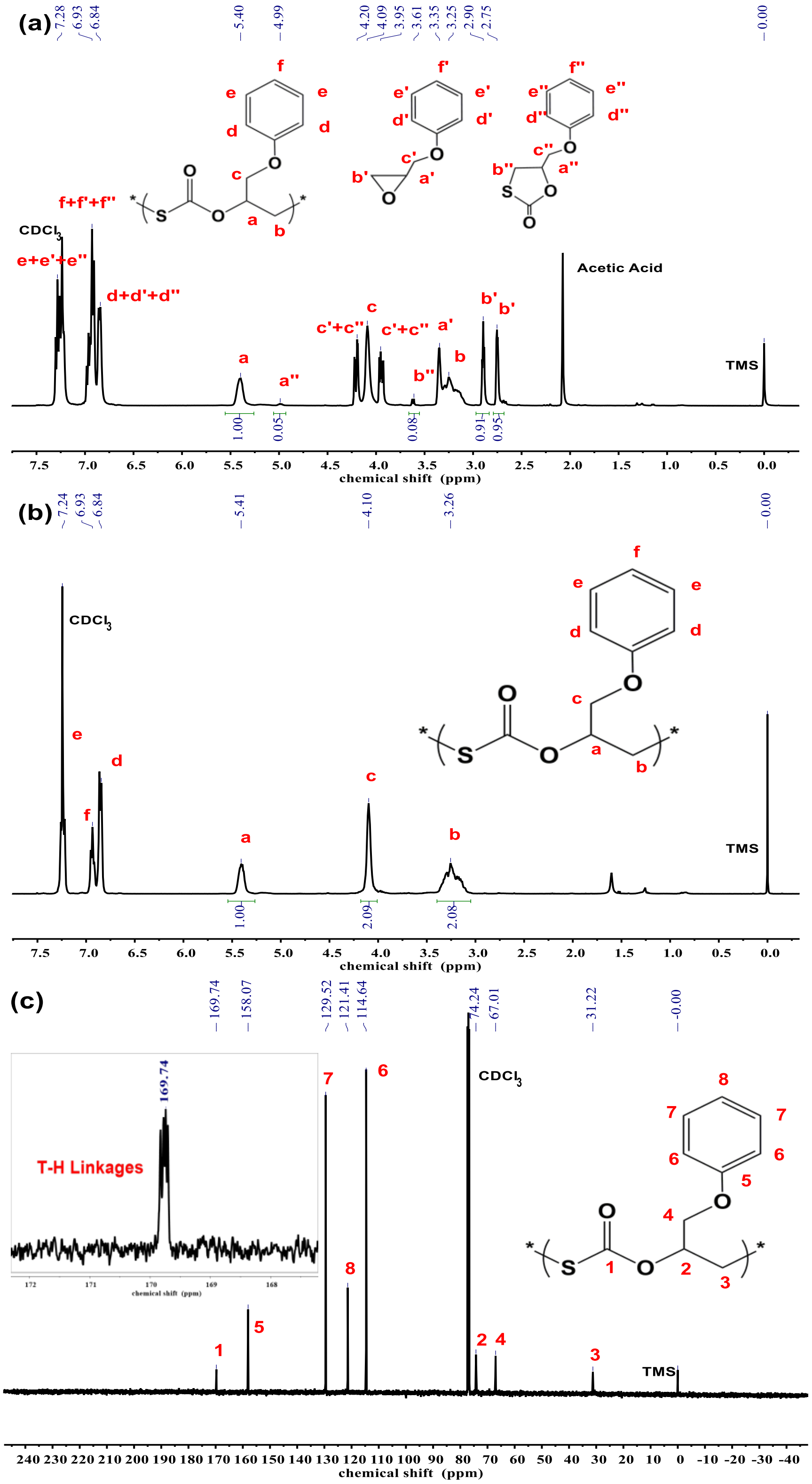

Supplementary Figure 29. (a)^{1}H NMR spectrum of the crude product of entry 3, Table 2; (b) ^{1}H NMR spectrum of the purified product of entry 3, Table 2; (c) ^{13}C NMR spectrum of the purified product of entry 3, Table 2.

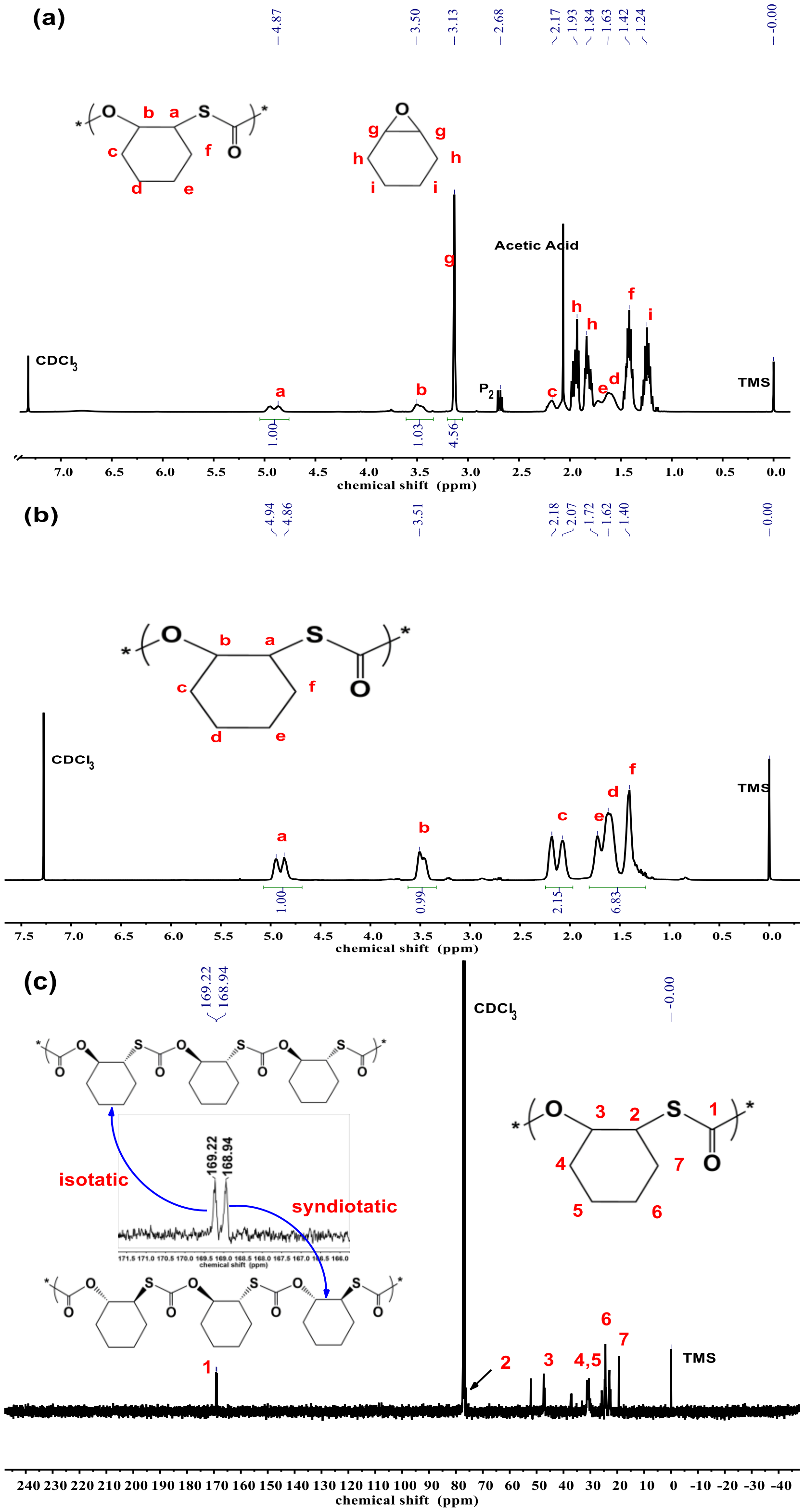

Supplementary Figure 30. (a)^{1}H NMR spectrum of the crude product of entry 5, Table 2; (b) ^{1}H NMR spectrum of the purified product of entry 5, Table 2; (c) ^{13}C NMR spectrum of the purified product of entry 5, Table 2.

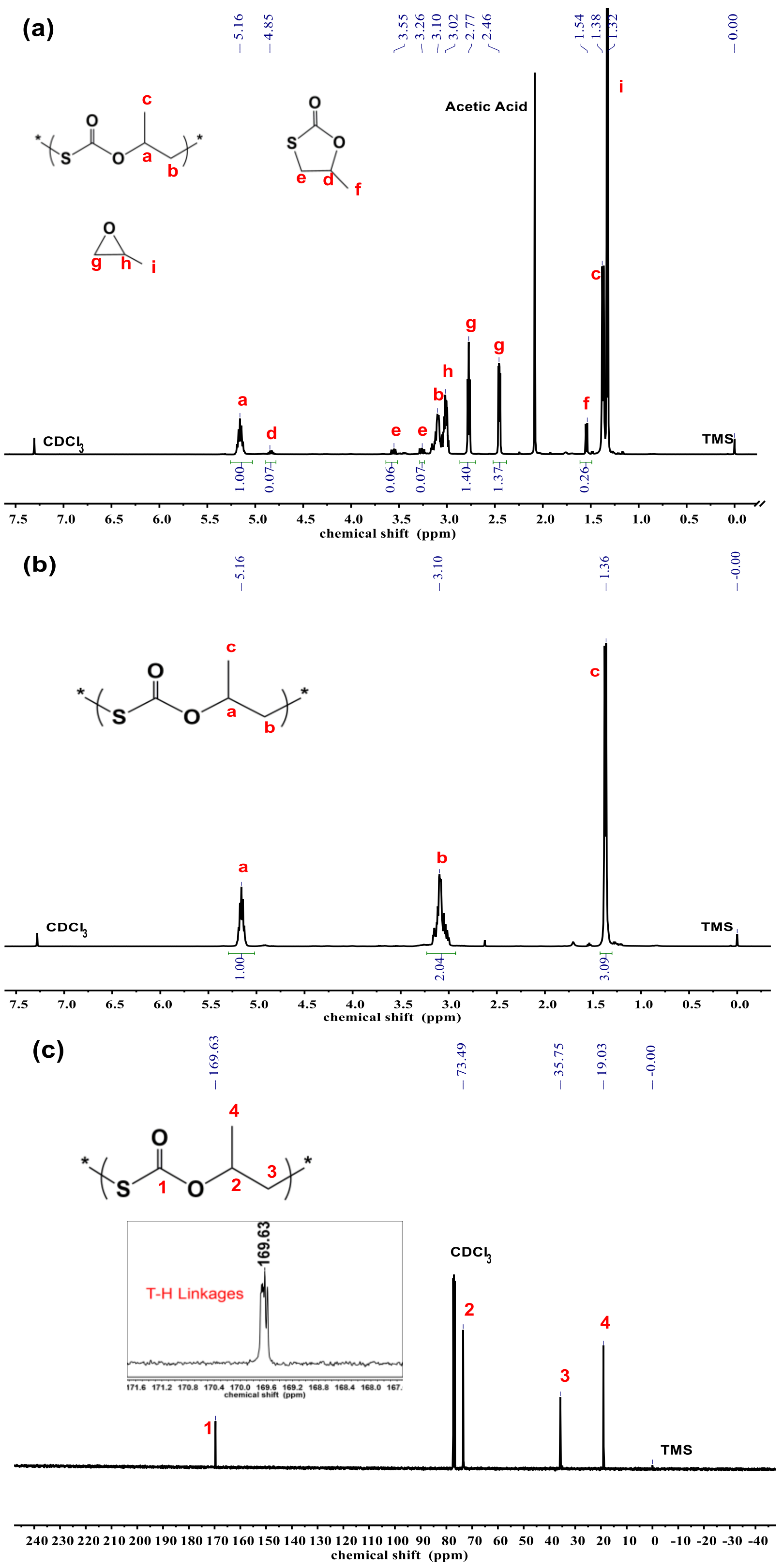

Supplementary Figure 31. (a)^{1}H NMR spectrum of the crude product of entry 1, Supplementary Table 1; (b) ^{1}H NMR spectrum of the purified product of entry 1, Supplementary Table 1; (c) ^{13}C NMR spectrum of the purified product of entry 1, Supplementary Table 1.

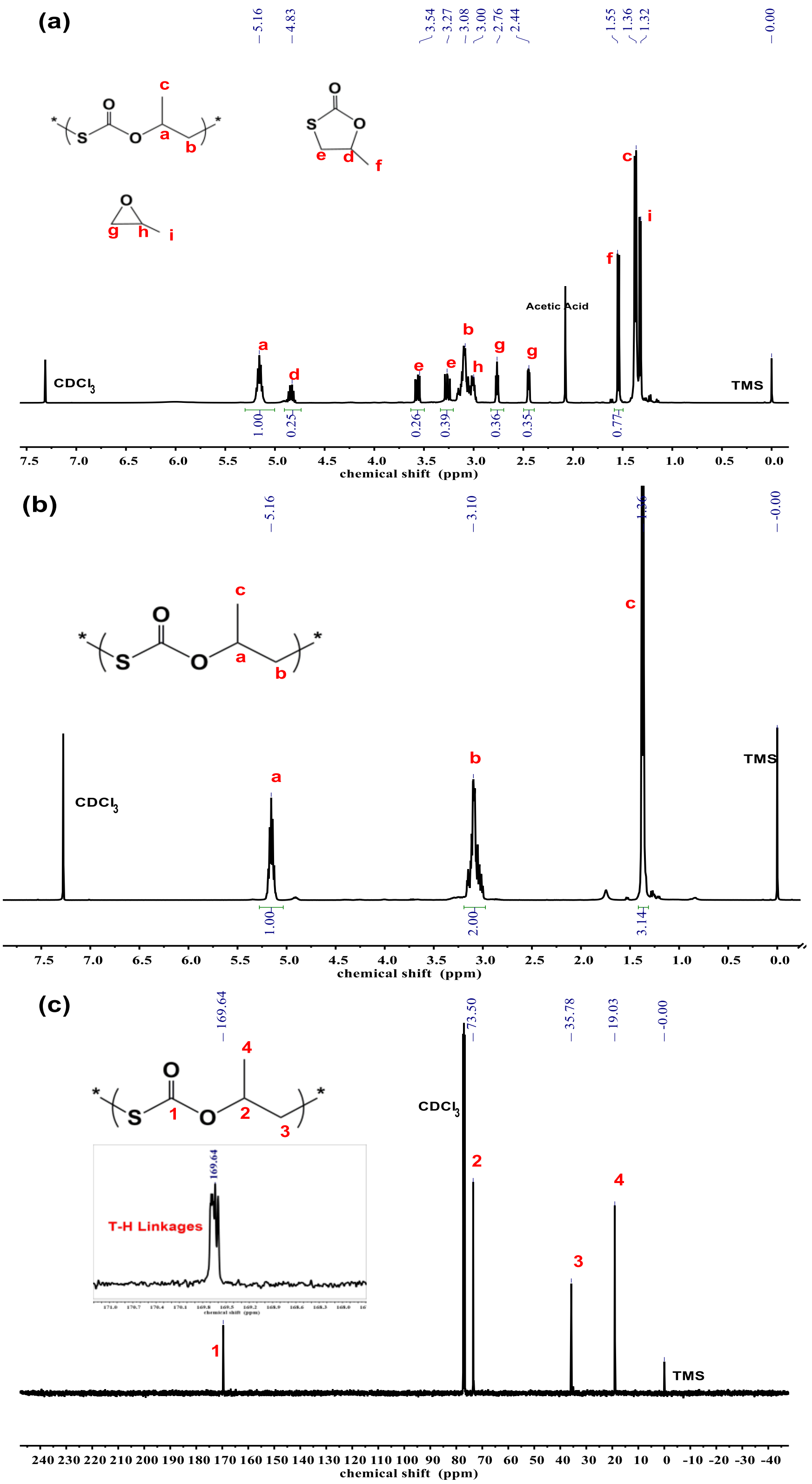

Supplementary Figure 32. (a)^{1}H NMR spectrum of the crude product of entry 3, Supplementary Table 2; (b) ^{1}H NMR spectrum of the purified product of entry 3, Supplementary Table 2; (c) ^{13}C NMR spectrum of the purified product of entry 3, Supplementary Table 2.

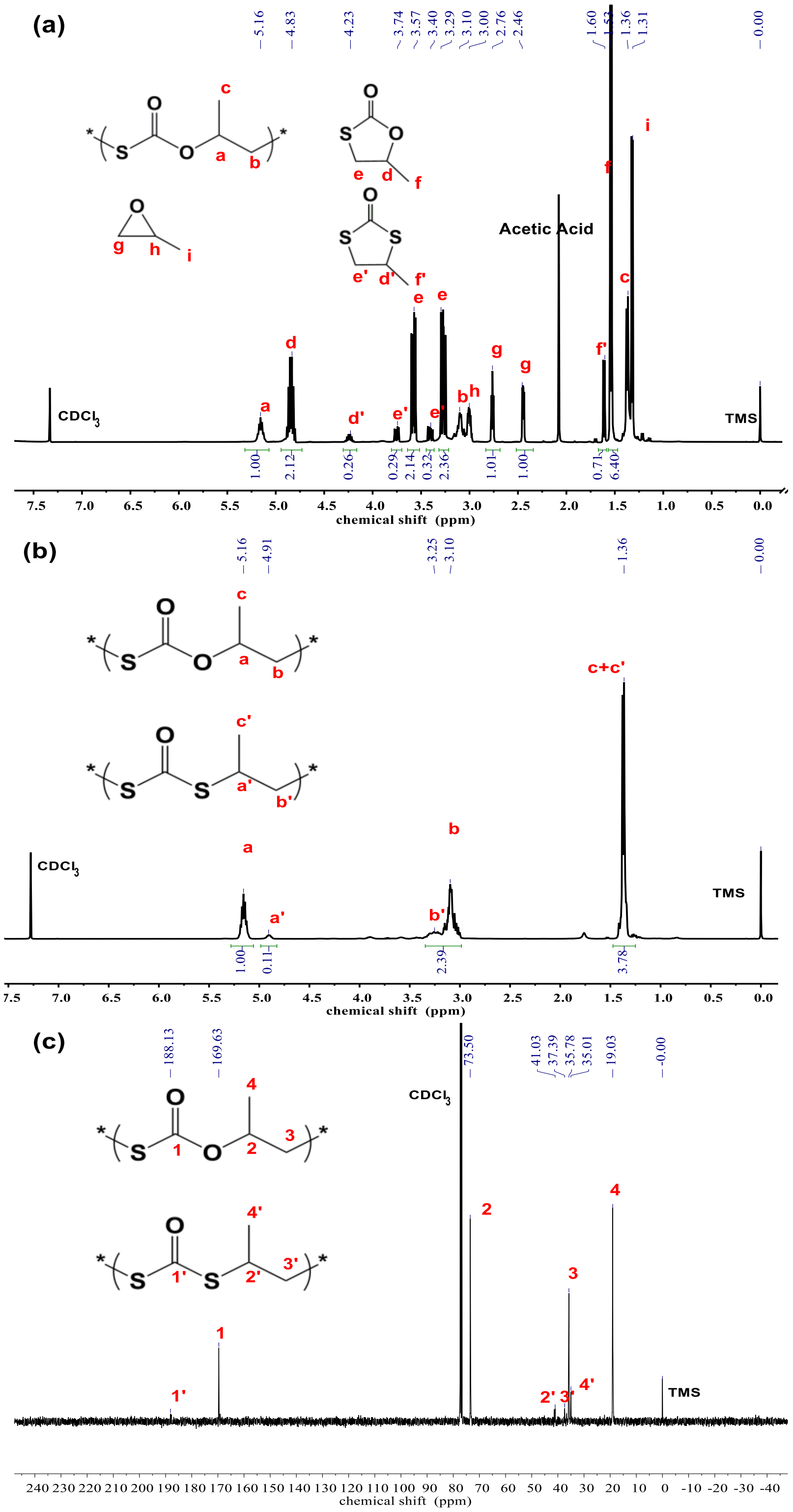

Supplementary Figure 33. (a)^{1}H NMR spectrum of the crude product of entry 4, Supplementary Table 2; (b) ^{1}H NMR spectrum of the purified product of entry 4, Supplementary Table 2; (c) ^{13}C NMR spectrum of the purified product of entry 4, Supplementary Table 2.

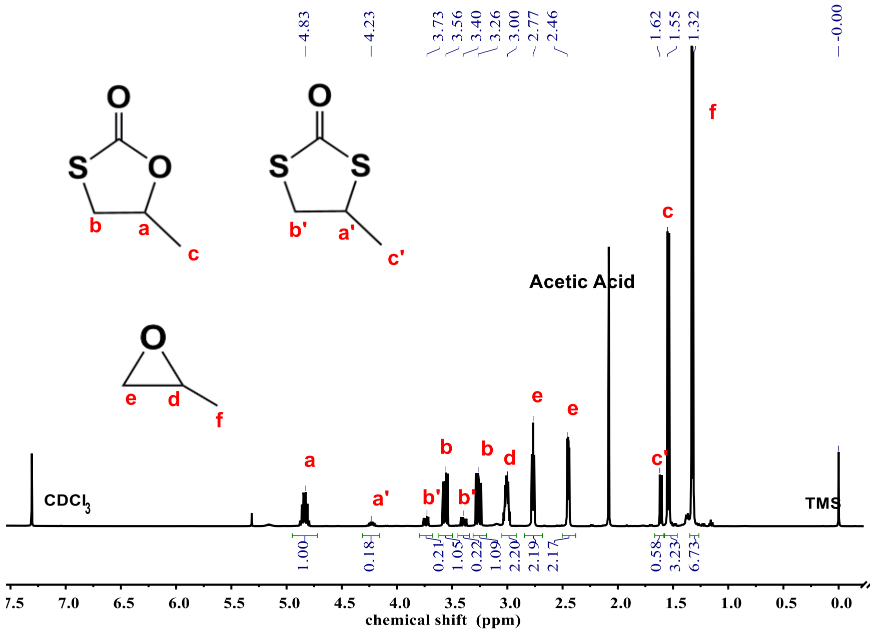

Supplementary Figure 34. ^1H NMR spectrum of the crude product of entry 6, Supplementary Table 2.

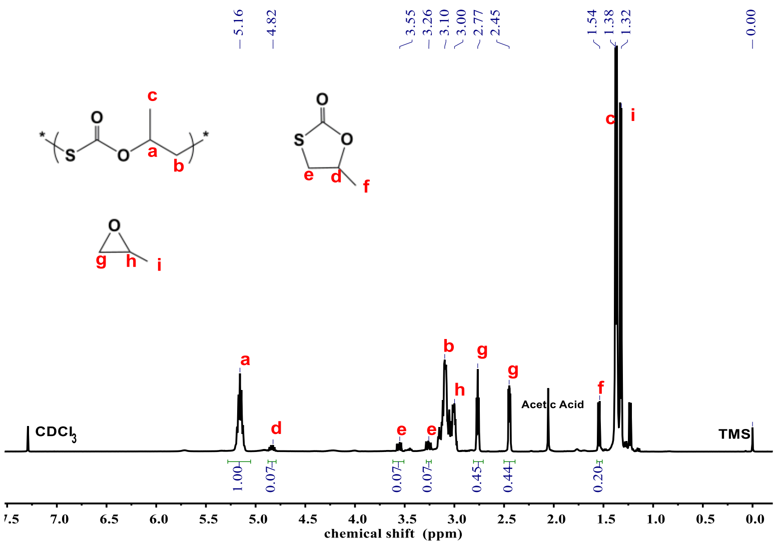

Supplementary Figure 35. ^{1}H NMR spectrum of the crude product of entry 11, Table 1.
